# The composition of piRNA clusters in *Drosophila melanogaster* deviates from expectations under the trap model

**DOI:** 10.1186/s12915-023-01727-7

**Published:** 2023-10-20

**Authors:** Filip Wierzbicki, Robert Kofler

**Affiliations:** 1 Institut für Populationsgenetik, Vetmeduni Vienna, Vienna, Austria; 2 Vienna Graduate School of Population Genetics, Vienna, Austria

**Keywords:** Transposable elements, piRNA clusters, *Drosophila melanogaster*, Trap model, Genome evolution, Population genetics

## Abstract

**Background:**

It is widely assumed that the invasion of a transposable element (TE) in mammals and invertebrates is stopped when a copy of the TE jumps into a piRNA cluster (i.e., the trap model). However, recent works, which for example showed that deletion of three major piRNA clusters has no effect on TE activity, cast doubt on the trap model.

**Results:**

Here, we test the trap model from a population genetics perspective. Our simulations show that the composition of regions that act as transposon traps (i.e., potentially piRNA clusters) ought to deviate from regions that have no effect on TE activity. We investigated TEs in five *Drosophila melanogaster* strains using three complementary approaches to test whether the composition of piRNA clusters matches these expectations. We found that the abundance of TE families inside and outside of piRNA clusters is highly correlated, although this is not expected under the trap model. Furthermore, the distribution of the number of TE insertions in piRNA clusters is also much broader than expected.

**Conclusions:**

We found that the observed composition of piRNA clusters is not in agreement with expectations under the simple trap model. Dispersed piRNA producing TE insertions and temporal as well as spatial heterogeneity of piRNA clusters may account for these deviations.

**Supplementary Information:**

The online version contains supplementary material available at 10.1186/s12915-023-01727-7.

## Background

Transposable elements (TEs) are short sequences of DNA that selfishly spread in host organisms, even if this selfish activity reduces the fitness of the host [1–3]. The ability to transpose within the host genome increases the chance of the TE to be transmitted to the next generation [3]. TEs are highly successful invaders that can be found in virtually all species investigated so far [4]. They show a large diversity in sequence, structure, and mechanisms for propagation [4, 5].

Although many examples of beneficial TE insertions have been reported [6], it is believed that most TE insertions are either neutral or deleterious [7, 8]. The fact that organisms with highly active TEs are frequently infertile strongly supports the idea of the deleterious effects of TEs [9–13]. Additionally, the observation that TEs are rare in coding regions but abundant in non-coding regions is thought to be largely due to negative selection against TEs [14–16]. Furthermore, the shift of the site-frequency-spectrum of TEs towards rare alleles is frequently interpreted as support for negative selection against TEs [17, 18]. The spread of TEs needs to be curbed as an unrestrained accumulation of deleterious TE insertions may drive host populations to extinction [19–21]. It was initially believed that TE invasions are controlled at the population level by negative selection against TEs [22, 23]. However, the discovery of small RNA-based host defence mechanisms profoundly changed our view and shifted the attention of many researchers from population genetic control of TE invasions to the functional implications of the host defence. In mammals and invertebrates, the host defence operates via piRNAs, small RNAs ranging in size from 23 to 29 nucleotides [24, 25]. These piRNAs mediate the repression of TEs at both the transcriptional and the post-transcriptional level [24–27]. Most piRNAs are produced from distinct source loci termed “piRNA clusters” which in total account for about 3.5% of the *Drosophila* genome [24]. Two distinct piRNA pathways operate in *Drosophila*, one in the germline and one in the soma, which mostly controls endogenous retroviruses that invade the germline via virus-like particles produced in the somatic tissue surrounding the germline [28, 29]. These two pathways rely on distinct sets of piRNA clusters. The germline pathway is based on dual-strand clusters while the somatic pathway primarily relies upon a single uni-strand cluster, *flamenco* [29]. piRNA clusters are largely found in pericentromeric regions [24, 30]. It was later discovered that some TE insertions outside of piRNA clusters are also able to generate piRNAs [31, 32]. Such dispersed piRNA producing source loci were found for many different TE families [31, 32]. The mechanism which converts TE insertions into turncoats, which support the host defence rather than the propagation of the TE, is based on maternally transmitted piRNAs [33–35]. Maternally inherited piRNAs bound to PIWI proteins mediate the installation of chromatin marks at TE insertions that are necessary for piRNA production [34, 35]. More recently, it was suggested that siRNAs may also drive the conversion of a TE insertion into a piRNA producing locus [36].

Under the current prevailing theory, the trap model, a TE invasion is stopped when a copy of the TE jumps into a piRNA cluster which then triggers the production of piRNAs that silence the TE [15, 29, 35, 37–39]. Several lines of evidence support the trap model. First, a single insertion in a piRNA cluster, such as X-TAS or 42AB, is able to silence a reporter [36, 40]. Second, an artificial sequence inserted into a piRNA cluster led to the production of piRNAs complementary to the inserted sequence [41]. Third, deletion of ZAM from the somatic piRNA cluster *flamenco* led to derepression of ZAM. Later the host reacquired the ability to suppress the TE likely due to a ZAM insertion in a germline cluster [42, 43]. Fourth, computer simulations showed that piRNA clusters are able to stop TE invasions, even in the absence of negative selection against a TE [20]. Fifth, studies monitoring TE invasions in experimental populations showed that piRNAs complementary to the newly invading TE rapidly emerged and that the generation of piRNAs was accompanied by the emergence of insertions in piRNA clusters [44, 45]. On the other hand, it was also shown that the observed number of cluster insertions at later generations, where the TE is likely silenced by the host, was lower than expected under the trap model [44, 45].

Additionally, computer simulations showed that piRNA clusters are solely able to control TE invasions if the clusters have a minimum size (as fraction of the genome) and that these minimum size requirement are barely met in some species [21]. Finally, deletion of three major piRNA clusters in the germline of *D. melanogaster* did not lead to an activation of TEs [46]. Due to these conflicting results, it is an important open question as to whether the trap model holds. Here, we argue that population genetics can shed light on this issue. Since TEs spread in populations, we argue that a complete understanding of TE invasions requires a synthesis of functional and population genetic considerations. Such a synthesis can lead to surprising outcomes. One notable example comes from the number of cluster insertions necessary to stop a TE invasion. Functional work suggests that a single TE insertion in a piRNA cluster may be sufficient to silence a TE [36, 40]. However, even when assuming that a single insertion is sufficient to stop a TE, population genetic models suggest that at least four insertions per diploid individual are necessary to stop a TE invasion. This can be explained by the fact that most TE insertions in piRNA clusters will be segregating in the population, and that recombination among these segregating cluster insertions will lead to a heterogeneous distribution of cluster insertions in the next generation, where some individuals will carry many cluster insertions and some solely a few or even none [20]. The TE will be active in the individuals without cluster insertions and thus the average number of cluster insertions in the population will increase. Only when individuals carry around four cluster insertions, do most individuals in the population end up with at least a single insertion. Interestingly, this requirement for four cluster insertions was robust over a wide range of different parameters and scenarios [20]. This stability in the number of cluster insertions required to silence a TE invasion led us to speculate that the composition of regions that act as transposon traps (e.g., possibly piRNA clusters) should differ markedly from regions that have no effect on TE activity. Such differences in the composition would provide us with an opportunity to test the trap model. We first performed computer simulations under the trap model and indeed found that the composition of transposon traps should differ from regions having no effect on TE activity in two important aspects. Firstly, for transposon traps, we do not expect a positive correlation between the abundance of TEs within and outside of the trap region, while such a correlation is expected for regions having no effect on TE activity. Secondly, we expect a narrow distribution of the abundance of different TE families in transposon traps.

By contrast, the expected distribution of TE insertions in regions having no effect on TE activity is much wider. Interestingly, the observed composition of piRNA clusters in five different *D. melanogaster* strains is not in agreement with expectations under the trap model.

Finally, we suggest amendments to the trap model that may account for the observed discrepancies. In particular, we think that dispersed source loci (DSL) and spatial or temporal heterogeneity of piRNA clusters may account for the observed composition of piRNA clusters.

## Results

It is an important open question whether TE copies inserting into piRNA clusters are responsible for stopping TE invasions (i.e., the trap model). Here, we argue that population genetics can shed light on this issue, as it makes testable predictions about the composition of regions that act as transposon traps (possible piRNA clusters). Notably, the composition of transposon traps should differ markedly from reference regions that have no effect on TE activity.

In this work, we proceed in two steps. First, we use simulations to identify key differences in the composition between regions that act as transposon traps (trap model) and reference regions that have no effect on TE activity (random model). Second, we test whether the observed composition of piRNA clusters in five *D. melanogaster* stains best fits with expectations under the trap model or the random model.

### Simulations of TE invasions

In a previous simulation study where we investigated TE invasions under the trap model, we realized that TE invasions are typically controlled when diploid individuals carry, on average, around four insertions in transposon traps (possible piRNA clusters), although we assumed that a single trap insertion per diploid is sufficient to silence the TE [20]. Recombination and random assortment among segregating trap insertions will lead to a distribution of trap insertions in populations, where some individuals will end up with several trap insertions and others with just a few or even none at all. Only when diploids carry an average of about four trap insertions, will the vast majority of the offspring end up with at least a single trap insertion. The observation that about four trap insertions per diploid individual are necessary to stop TE invasions was highly robust over all evaluated parameters (e.g., different sizes of genomes and piRNA clusters, transposition rates and population sizes; [20]. Based on this robustness of the number of trap insertions, we hypothesized that the composition of transposon traps should deviate from the composition of reference regions having no effect on TEs in two aspects. First, the distribution of the number of insertions for all different TE families should be very narrow in transposon traps (most families should have around 2 insertions in transposon traps per haploid genome) while the distribution should be much broader for reference regions. Second, for the different TE families, the abundance of TEs in reference regions and the rest of the genome should be highly correlated whereas no correlation is expected between the TE abundance in transposon traps and the rest of the genome.

**Fig. 1:**
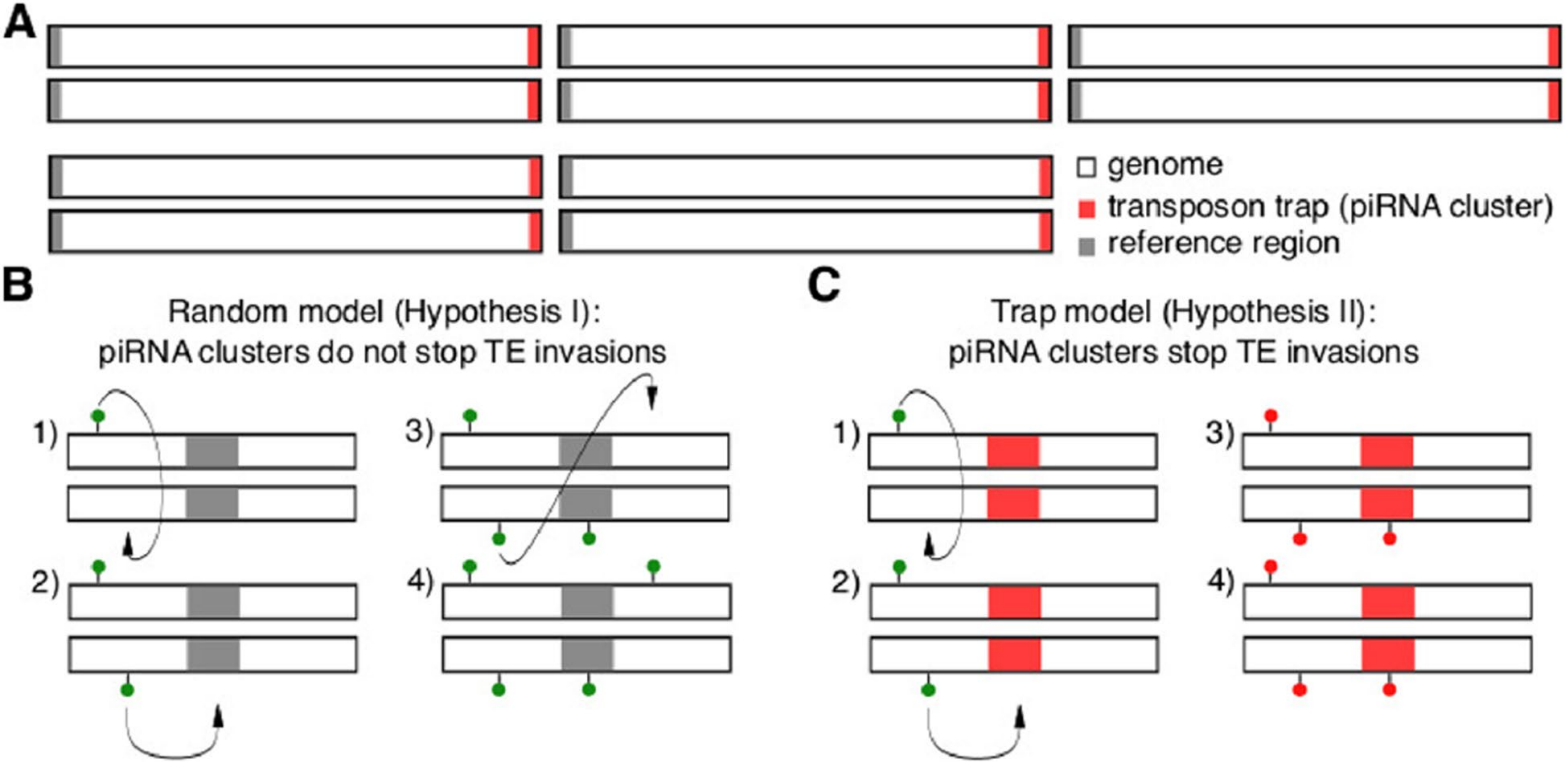
Overview of our simulation approach for testing the trap model. **A** We simulated diploid organisms with 5 chromosomes and a uniform recombination rate of 4 cM/Mb. Equally sized transposon traps and reference regions were simulated on opposite ends of the chromosomes. In our model, we assumed that a TE insertion into a reference region (**B**) has no effect on TE activity while a single insertion into a transposon trap (**C**) silences the TE [37, 40]. The numbers 1 to 4 refer to successive time points during a TE invasion. Green circle: active TE insertion, red circle: inactive TE insertion

**Fig. 2:**
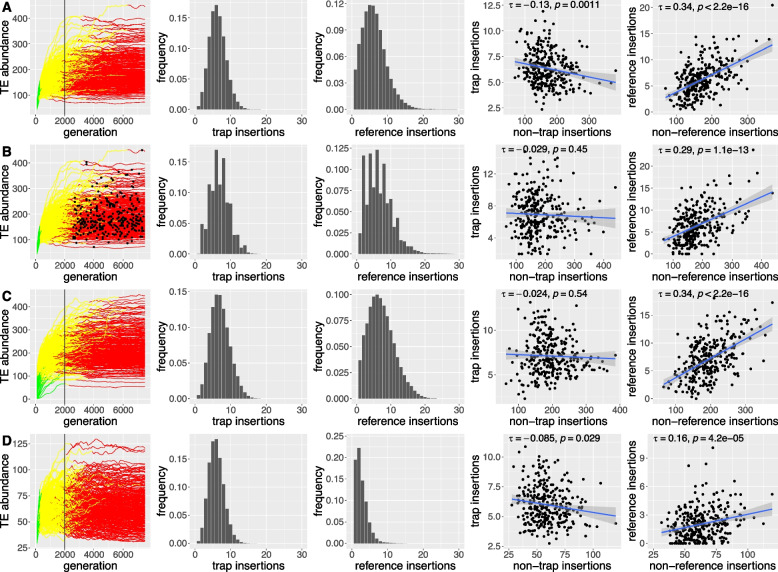
Key differences in the composition of transposon traps (possible piRNA clusters) and reference regions under four different models (**A**–**D**). We simulated 300 replicates for each model (each line is a replicate). From left to right, panels show the TE abundance during invasions, where colors indicate the three distinct phases of TE invasions (invasions controlled by TE insertions in transposons traps are shown in yellow and red; [20]) (i), a histogram with the abundance of TE insertions in transposon traps (ii), reference regions (iii), and the correlation between the abundance of TE insertions in transposon traps (iv), or reference regions (v) and the rest of the genome. **A** A simple model with neutral TE insertions and a constant transposition rate ($u=0.1$). Invasions were sampled at generation 2000 (black line). **B** A simple model with neutral TE insertions and constant transposition rate ($u=0.1$). Invasions were sampled at different time points between 2500 and 7500 generations (black dots). **C** A model with neutral TE insertions. Each replicate has a different, randomly chosen, transposition rate ($0.005 \le u \le 0.5$). **D** A model where TE insertions have negative fitness effects (site-specific negative effects: 10% of the insertions each have $x=0.1$, $x=0.01$, $x=0.001$, and $x=0.0001$; 60% are neutral). All replicates have a constant transposition rate ($u=0.1$)

We performed extensive simulations of TE invasions with Invade [20] to validate our hypotheses about these two key differences in the composition of transposon traps and reference regions. The choice of parameters for the simulations was inspired by *D. melanogaster*. We simulated 5 chromosome arms with a uniform recombination rate of 4 cM/Mb. On one end of each chromosome we simulated transposon traps and on the other end reference regions (Fig. 1A). While TE insertions into reference regions do not inactive the invading TE (Fig. 1B), insertions into traps inactive all TE copies in a given individual (Fig. 1C). Both the transposon traps and the reference regions each cover 3.5% of the genome, similar to dual-strand piRNA clusters in the germline of *D. melanogaster* [24]. We assumed a constant transposition rate *u* (i.e., the probability that a single TE copy generates a new copy in the subsequent generation) and novel TE insertions were distributed randomly in the genome. We simulated a population size of $N=1000$ and non-overlapping generations. To avoid the stochastic early stages of an invasion, where a novel TE is frequently lost by genetic drift [20, 47], we triggered each invasion by introducing 1000 TE insertions at random genomic positions (starting frequency ${f= 1/(2\cdot 1000)}$). For each scenario we simulated 300 replicates. Note that replicates of TE invasions may either be interpreted as invasions of the same TE family in different populations (species) or as invasions of different TE families in the same population. In this work we rely on the second interpretation, which allows us to link our simulation results to the observed abundance of the different TE families in piRNA clusters (see below).

In agreement with recommendations for biological modeling [48], we started with a simple model and then gradually increased the complexity. In the first and simplest scenario, we simulated an identical transposition rate in all replicates ($u=0.1$) and neutral TE insertions (Fig. 2A). At generation 2000 we measured the abundance of TEs in (i) the genome, (ii) the transposon trap, and (iii) the reference region. Note that by generation 2000 the invasion is silenced in all replicates, either by segregating or fixed insertions in transposon traps. In a previous study we referred to these distinct stages of TE invasions under the trap model as the shotgun phase and inactive phase, respectively ([20]; Fig. 2, left panels, yellow and red). Even under this simple model we observed a striking heterogeneity in the abundance of TE insertions during the invasion among the 300 replicates (Fig. 2A).

We first investigated the distribution of the TE abundance in both the transposon traps and reference regions. In agreement with our hypothesis, we found that the distribution of the TE abundance in transposon traps is narrower than that of the reference regions (Fig. 2A, second vs third panel). Very few individuals from these 300 replicate populations have less than 1 (0.06%) or more than 14 (0.07%) TE insertions in a transposon trap (Fig. 2A, second panel). By contrast, more individuals have less than 1 (2.00%) or more than 14 (1.97%) insertions in reference regions. Importantly, this observation is independent of the size of transposon traps and reference regions (Additional file 1: Fig. S1). While it is obvious that individuals with silenced TEs will have at least one insertion in a transposon trap, it is perhaps less clear why more than 14 trap insertions are also not expected under the simple trap model. Since a single TE insertion in a transposon trap silences an invading TE, a continuous accumulation of insertions in the trap regions is not feasible. Only recombination among segregating trap insertions can lead to a slightly elevated number of trap insertions in diploid individuals [20]. However, the TE distribution resulting from recombination in the transposon traps will be narrower than in reference regions, where in addition to recombination, multiple independent TE insertions may occur (Fig. 2A).

Second, we investigated the correlation of the TE abundance between transposon traps or reference regions and the rest of the genome. Again in agreement with our hypothesis, we observed a significant positive correlation between the abundance of TEs in reference regions and the rest of the genome but not between transposon traps and the rest of the genome (Fig. 2A; fourth and fifth panel).

It is obvious that the abundance of TE families in the genome and a random sample of the genome (i.e., the reference region) will correlate. However, the same relation does not hold for transposon traps, since any TE insertion in the trap will deactivate the TE, thus preventing further accumulation of TEs by transposition. For this reason transposon traps consistently accumulate about 2–3 TE insertions per haploid genome during TE invasions, irrespective of the simulated scenario (different trap sizes, transposition rates, genome sizes, population sizes [20]).

Next, we aimed to investigate the robustness of those two key differences between transposon traps and reference regions in more complex models. In our simple scenario we assumed that all replicates are sampled at the same time (i.e., 2000 generations after the invasion was triggered). It may, however, be argued that different TE families in an organism (corresponding to replicates in the simulations) are usually captured at different stages of the life cycle of a TE. For example, the P-element invaded *D. melanogaster* populations within the last century, while non-LTR TEs likely invaded thousands of years ago [49–51]. To address this issue, we randomly sampled TE invasions between generation 2500 and 7500 (Fig. 2B; black dots in the left panel). We did not sample any invasion at the early stages, where the TE is not yet controlled by insertions in transposon traps (Fig. 2B; left panel, green; rapid invasion phase [20]). Our two key differences between transposon traps and reference regions were robust to variation in the sampling time of TE invasions (Fig. 2B).

So far, we have assumed that all replicates (interpreted as different TE families) have an identical transposition rate. It is, however, likely that different TE families have different transposition rates [44, 52, 53]. Although, the influence of the transposition rate on TE copy numbers is likely small under the trap model, variation in the transposition rate could lead to an accumulation of different TE copy numbers during invasions [20, 54, 55]. To address this, we randomly selected different transposition rates (between $u=0.005$ and $u=0.5$) for each replicate (Fig. 2C). Our two key differences between transposon traps and reference regions were robust to variation in the transposition rate (Fig. 2C).

Above, we only considered neutral TE insertions, i.e., insertions that have no effect on host fitness. While this may be true for many TEs, it is likely that at least some TE insertions have deleterious effects to the fitness of the host [7, 8]. Therefore, in the next model we considered negative effects of TE insertions (*x*) using the fitness function $w=1-n{\cdot }x$ (*w* fitness, *n* number of TE insertions). For example, an individual that carries 2 TE insertions with negative effects of $x=0.1$ has, on average, 20% less offspring than an individual without any TE insertions. Initially, we simulated a scenario where all TE insertions have an equal constant effect. To avoid an unlikely equilibrium state between transposition, selection, and piRNA clusters (TSC balance [20]), we assumed that TE insertions in transposon traps are neutral. We performed 300 simulations for each of the following negative effects: $x=0.0001$, $x=0.001$, and $x =0.01$ (Additional file 1: Fig. S2A–C). As expected, with weak negative effects ($x=0.0001$; Additional file 1: Fig. S2A), the invasions resemble the neutral scenario (Fig. 2A; $N{\cdot }x<1$). Large negative effects ($x=0.001$, $x=0.01$) had a notable impact on the abundance of TEs during the invasions (Additional file 1: Fig. S2B, C). While negative selection had a minimal effect on the abundance of TEs in trap regions, the abundance in reference regions was markedly reduced, with many individuals having zero insertions in reference regions (Additional file 1: Fig. S2B, C). Furthermore, we found a positive correlation between the TE abundance in the genome and reference regions but not with transposon traps (where the correlation was actually negative; Additional file 1: Fig. S2). Even when we further relax our assumptions by considering negative selection against all TE insertions, including insertions in transposon traps, our two key differences are robust (Additional file 1: Fig. S3). Note that invasions with strong negative selection reach TSC balance and is thus neither stopped by segregating nor fixed insertions in transposon traps (Additional file 1: Fig. S3C; [20]).

Thus far, we have assumed that all TE insertions have an equal negative effect on host fitness, irrespective of the genomic insertion site. It is, however, possible that different TE insertions have diverse fitness effects depending on the insertion site ([8, 56]). For example, insertions into coding sequences are likely more harmful than insertions in intergenic regions. We evaluated the effect of heterogeneous fitness effects of TE insertions using the linear fitness function: $w=1-\sum _{{i=1}}^{{n}}{{x}_i}$ where ${x}_i$ is the negative effect of each TE insertion. With such a site-specific model, we may vary (i) the effect size of the TE insertions and (ii) the proportion of the genome at which a TE insertion will lead to the given negative effects. We varied the fraction of sites where a TE insertions causes negative fitness effects ($x=0.01$) from 10 to 70%. The remaining 90 to 30% of the possible insertion sites were neutral.

Our two key differences between trap and reference regions were robust to variation in the number of neutral insertion sites (Additional file 1: Fig. S4). Next, we considered a more complex distribution of site-specific deleterious effects of TE insertions. We assumed that 10% of the insertions each have a negative effect of $x=0.1$, $x=0.01$, $x=0.001$, and $x=0.0001$, while the remaining 60% of the sites were neutral (Fig. 2D). Our key differences were again robust (Fig. 2D).

Up to this point, we have simulated TE insertions with identical fitness effects among replicates (corresponding to TE families). It could, however, be argued that different TE families have diverse fitness effects. For example, a TE family with an insertion bias into promotor regions may be, on average, more deleterious than a TE family that has an insertion preference for intergenic regions. In agreement with this, previous work suggests that negative effects of TE insertions may vary among TE families [18, 23]. To consider such a scenario, we performed simulations with different negative effects. For each replicate, we randomly picked a different negative effect between $x=0.001$ and $x=0.1$ for 40% of the sites in the genome while insertions into the remaining 60% were neutral. Within a replicate, all non-neutral TE insertions had the same negative effect (Additional file 1: Fig. S5). Under this scenario, we once again found a narrow distribution of the TE abundance within traps and no correlation of the TE abundance between trap regions and the rest of the genome (Additional file 1: Fig. S5).

In summary, we identified two key differences in the composition of transposon traps and reference regions that were robust in all evaluated scenarios. If piRNA clusters act as transposon traps, we expect (i) no positive correlation between the abundance of TEs in piRNA clusters and the rest of the genome and (ii) a narrow distribution of the abundance of TE insertions in piRNA clusters, with few individuals having less than 1 or more than 14 cluster insertions for a given TE family.

### The TE composition of piRNA clusters is not in agreement with expectations under the trap model

We next asked whether the observed composition of piRNA clusters is more in agreement with expectations under the trap or the random model, based on the two key differences between transposon traps and reference regions identified above. To address this question, we investigated the TE composition in five *D. melanogaster* strains (Canton-S, DGRP-732, Iso-1, Oregon-R, and Pi2). For all five strains, Illumina paired-end reads and genome assemblies are publicly available ([57–62]). We ensured that the assemblies are of high quality, having complete assemblies of most piRNA clusters (Additional file 1: Table S1). Based on the number of completely assembled piRNA clusters, the five assemblies analyzed in this work are among the best out of 37 high-quality (mostly based on long reads) assemblies of diverse *D. melanogaster* strains [63, 64] (Additional file 1: Table S2). Ovarian small RNA data are available for Canton-S, DGRP-732, Iso-1, and Oregon-R ([49, 65, 66]). For this work, we generated ovarian small RNA data for Pi2. We employed three complementary approaches to estimate the composition of piRNA clusters in these five strains. First, we identified TE insertions in piRNA clusters based on paired-end reads aligned to the *D. melanogaster* reference genome (release 5; [67, 68]). Based on the standard annotations of piRNA clusters [24] and our tool PoPoolationTE2, which locates TE insertions in a reference genome using paired-end data, TE insertions in piRNA clusters and the rest of the genome were identified [69]. This approach has the advantage that it relies on the widely used standard annotations of piRNA clusters but the disadvantage that some TE insertions in piRNA clusters may be missed. Since piRNA clusters are evolving rapidly [30, 46], the sequences of piRNA clusters in the five strains may have diverged from the reference genome. Therefore, some strain-specific reads may not align to the reference genome and thus not all TE insertions may have be identified. Second, we identified TE insertions in the assemblies of the investigated strains using RepeatMasker [70] and performed a lift-over of the annotations of piRNA clusters from the reference genome to the assemblies with our CUSCO approach, where the positions of piRNA clusters are identified using unique sequences flanking the reference clusters [62]. In addition to clusters being flanked by two unique sequences, we also included telomeric piRNA clusters into the analysis (e.g., X-TAS) [40, 71]. This approach has the advantage that the composition of the rapidly evolving piRNA clusters may be more accurately captured as we rely on assemblies of the investigated strains. Furthermore, this approach is based on the widely used standard annotations of piRNA clusters in *D. melanogaster* [24]. The disadvantage is that the positions of some reference clusters cannot be identified in the assemblies (for some reference clusters unique flanking sequences could not be identified and some flanking sequences cannot be mapped to the assemblies). Finally, it is possible that, in addition to the composition of piRNA clusters, the location of piRNA clusters is also evolving rapidly [46]. To address this issue, we employed a third approach, where we performed a de novo annotation of piRNA clusters in the assemblies of the five strains. We used strain-specific small RNA data for the annotation of piRNA clusters. TE insertions were again identified with RepeatMasker [70]. This approach has the benefit that strain-specific variation in both, the location and the composition of piRNA clusters is taken into account, but it has the downside that the locations of these clusters have not yet been substantiated by complementary approaches. For example, apart from an enrichment of piRNAs, dual-strand clusters of the germline typically also show an enrichment of H3K9me3 methylation marks and of Rhino and Kipferl binding sites [32, 72]. This information is not yet available for the de novo annotated piRNA clusters. For all three complementary approaches, we only considered TE families being active in the germline.

**Fig. 3:**
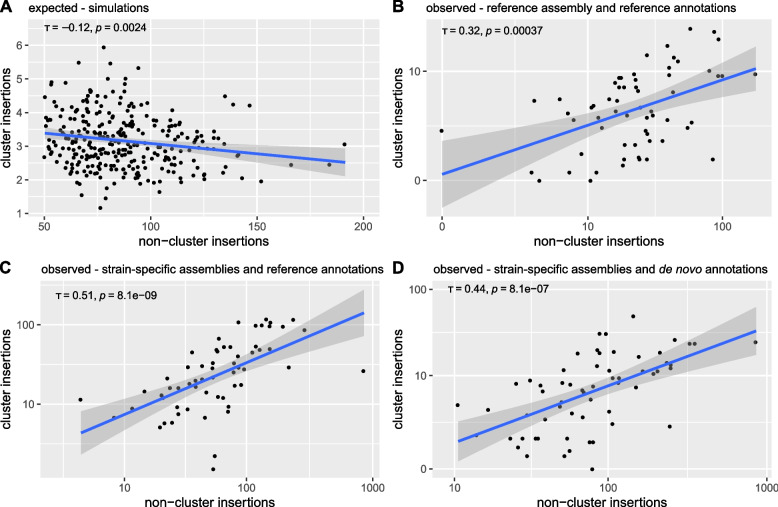
Expected and observed correlation between the TE abundance in piRNA clusters and the rest of the genome. **A** Expected correlation based on simulations under the trap model (neutral insertions and $u=0.1$). **B** Observed correlation based on short reads aligned to the reference genome and the reference annotations of piRNA clusters. **C** Observed correlation based on strain-specific assemblies and a lift-over of the reference annotations of piRNA clusters using unique sequences flanking the clusters. **D** Observed correlation based on strain-specific assemblies and de novo annotations of piRNA clusters. All counts refer to copy numbers per haploid genome. For the observed data we averaged the counts over the five strains. Solely TE families active in the germline were considered. Kendall rank correlation coefficients are reported

**Fig. 4:**
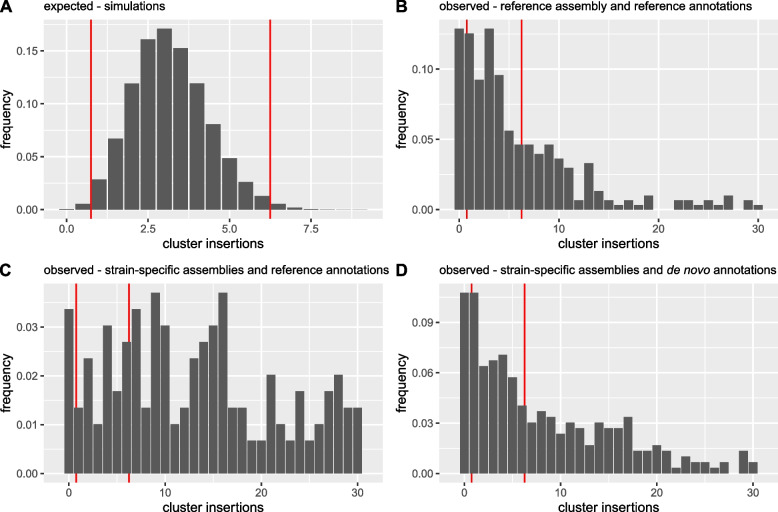
Expected and observed abundance of different TE families in piRNA clusters. **A** Histogram showing the expected abundance based on simulations under the trap model (for haploid genomes). **B** Observed abundance based on short reads aligned to the reference genome and the reference annotations of piRNA clusters. **C** Observed abundance based on strain-specific assemblies and a lift-over of the reference annotations of piRNA clusters, using unique sequences flanking the clusters. **D** Observed abundance based on strain-specific assemblies and de novo annotations of piRNA clusters. All counts refer to copy numbers per haploid genomes. For the observed data, we averaged the abundance in the five investigated strains. Only TE families active in the germline were considered. At least 98% of the simulations under the trap model are between the red lines. The *x*-axis was truncated at 30 insertions

We first examined the correlation between the abundance of different TE families in piRNA clusters and the rest of the genome (Fig. 3). Under the trap model, we expect either no correlation or a negative correlation (Figs. 2 and 3A) whereas under the random model a positive correlation is expected (see above).

We found a positive correlation between the average number of TE insertions in piRNA clusters and the rest of the genome, with all three approaches for quantifying the TE abundance in the five *D. melanogaster* strains (Fig. 3B, C, D). This correlation can also be found if each strain is analyzed separately (Additional file 1: Fig. S6).

In both assembly-based approaches, the TE copy numbers are estimated with RepeatMasker, which occasionally provides fragmented annotations for highly diverged TE insertions or TEs with internal deletions. It may be argued that such fragmented TE insertions could lead to wrong correlations between the TE content in clusters and the rest of the genome. To address this issue, we repeated the analysis using two different approaches. We first merged fragmented TE annotations with the tool Onecodetofindthemall.pl [73] and again found a correlation between the TE abundance within and outside of piRNA clusters (Additional file 1: Fig. S7A; based on the assemblies and reference clusters). Second, we used a highly conservative approach solely considering contiguous full-length insertions and again found the correlation between the TE abundance within and outside of piRNA clusters (Additional file 1: Fig. S8A; based on the assemblies and reference clusters). Finally, gaps in the assemblies of piRNA clusters indicate assembly problems [62]. Therefore, we repeated the analysis by excluding clusters with gaps but again found a significant correlation between the TE abundance within and outside of piRNA clusters (Additional file 1: Fig. S9A).

Next, we focused on the abundance of the different TE families in piRNA clusters. Our simulations show that the abundance of TE families in transposon traps should follow a narrow distribution, with no family having less than 1 and only a few having more than 14 insertions (Figs. 2 and 4A). Based on our three complementary approaches, we estimated the abundance of each TE family per haploid genome in the five strains. We found that the observed distribution of the TE abundance in piRNA clusters differs substantially from expectations under the trap model (Fig. 4). First, several TE families do not have a single cluster insertion (Fig. 4B, C, D). For example, we could not find cluster insertions for the R2-element, Tirant, Bari1, flea, and jockey in some strains (Additional file 1: Table S3). Second, many families have many more insertions in piRNA clusters than expected (Fig. 4B, C, D).

As mentioned above, the assembly-based approaches rely on RepeatMasker, which occasionally provides fragmented annotations for TE insertions. Such fragmented annotations could boost the number of TE insertions in piRNA clusters causing the observed over-representation of some TE families in piRNA clusters. To address this issue, we repeated the analysis by merging fragmented annotations with the tool Onecodetofindthemall.pl [73] and again found an over-representation of several TE families in piRNA clusters (Additional file 1: Fig. S7B). This over-representation of TEs in piRNA clusters is also found when we just consider full-length insertions of TEs or piRNA clusters assembled without gaps (Additional file 1: Figs. S8B, S9B). This analysis also revealed that many TE families (27.3%) do not have a single full-length insertion in a piRNA cluster (Additional file 1: Fig. S8B).

To summarize, we find a correlation between the abundance of TEs in piRNA clusters and the rest of the genome, contrary to expectations under the trap model. Moreover, we observed that many TE families either do not have a single insertion in a piRNA cluster or have a larger than expected number of cluster insertions. These observations are more consistent with the random model, which assumes that TE insertions in piRNA clusters have no effect on TE activity.

### Abundance of dispersed piRNA producing TE insertions

**Fig. 5:**
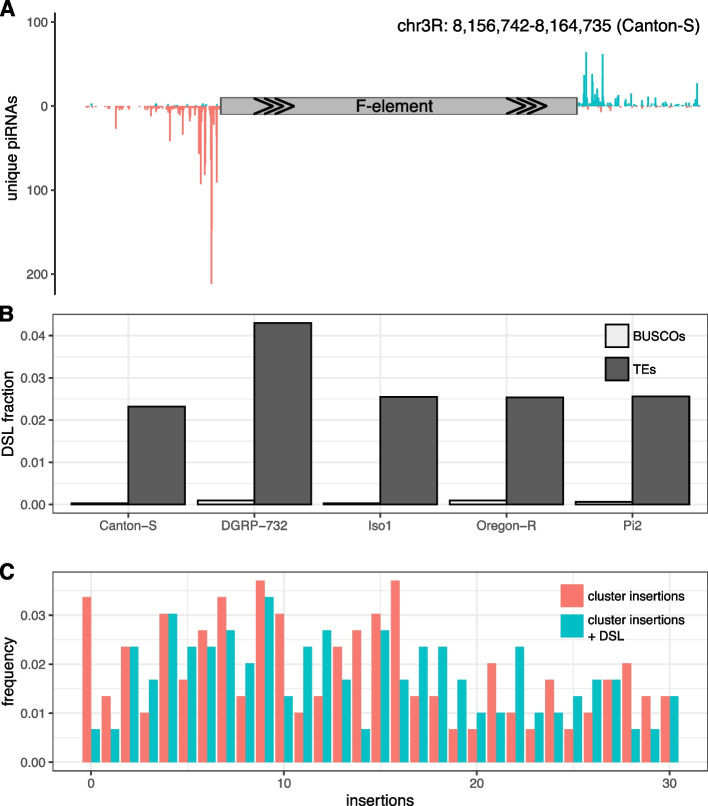
DSL could compensate for the missing cluster insertions. **A** Example of a DSL in Canton-S. Note the typical signature of DSL where antisense piRNAs (red, negative *y*-axis) align upstream of the TE insertions (F-element) and sense piRNAs (blue, positive *y*-axis) downstream of the TE [31]. **B** Abundance of DSL in the five investigated strains (dark gray). As a negative control we also computed the fraction of BUSCO genes with a typical DSL signature (light gray; BUSCO genes should not have DSL signatures). **C** Abundance of piRNA producing loci in the five strains. Data are shown for cluster insertions (red) as well as cluster insertions plus DSL (cyan). Note that the number of families without piRNA producing locus is dramatically reduced when DSL are considered

For several TE families, we did not find a single cluster insertion, which is unexpected if piRNA clusters control TE invasions. Apart from assembly problems (see the “Discussion” section), there is an alternative hypothesis which may account for the missing cluster insertions. Recently, Gebert et al. [46] showed that three major piRNA clusters can be deleted with no effect on the activity of the TEs. They suggest that this is due to redundancy in the host defence, where dispersed TE insertions may also produce piRNAs. These DSL could compensate for the missing cluster insertions. TE families without cluster insertions should thus have at least one DSL. Such DSL have a distinct piRNA signature that can be recognized in the genome. piRNA production frequently extends from the TE into the genomic regions flanking the TE insertion, such that antisense piRNAs are produced upstream of the TE and sense piRNAs downstream of the TE [31]. To identify DSL, we scanned the assemblies of the five strains for TE insertions flanked by these asymmetric piRNA signatures (for example, Fig. 5A). To evaluate the performance of our algorithm for finding DSL, we computed the fraction of conserved genes (BUSCO genes) with asymmetric piRNA signatures. Since none (or almost none) of the conserved BUSCO genes are expected to act as a piRNA producing locus, this approach provides us with an estimate of the fraction of false positive DSL identified by our approach. We found very few BUSCO genes with such asymmetric piRNA signatures and therefore argue that our approach has a high specificity (Fig. 5B). Using our approach, we estimate that about $2-5\%$ of the TE insertions are piRNA source loci outside of piRNA clusters (Fig. 5B). The abundance of DSL varies among the TE families and the strains (Additional file 1: Fig. S10). DSL were more evenly distributed along chromosomes than insertions in piRNA clusters, which were most abundant near centromeres (Additional file 1: Fig. S11). Finally, we asked whether the DSL could compensate for the missing TE insertions in piRNA clusters. Indeed, we found a DSL for most of the TE families not having a single cluster insertion (Fig. 5 C, Additional file 1: Fig. S12, Table S3; based on the strain-specific assemblies and the reference clusters). Only Tirant and Bari1 in Oregon-R do not have a single piRNA producing locus (neither cluster insertion nor DSL; Additional file 1: Table S3). TE families with and without cluster insertions have similar ping-pong signatures, i.e., a typical 10nt overlap between sense and antisense piRNAs resulting from an active piRNA pathway, suggesting that TE families with any piRNA producing locus (either DSL or cluster insertion) are silenced by the piRNA pathway (Wilcoxon rank sum test with *Z*-scores $W = 1874$, $p = 0.096$; Additional file 1: Fig. S13, Table S4; [24, 25]).

In summary, our results show that we find at least one piRNA producing locus, either a cluster insertion or a DSL, for most of the recently active TEs in *D. melanogaster*. Therefore, DSL can largely compensate for the missing cluster insertions of some TE families.

## Discussion

In this work, we showed that the observed composition of piRNA clusters in five *D. melanogaster* strains is not in agreement with expectations under the simple trap model, i.e., the notion that a single insertion in a piRNA cluster stops the proliferation of TEs [37, 40].

Based on extensive simulations of TE invasions under the trap model, we identified two key differences between genomic regions where a TE insertion represses TE activity and regions where insertions have no effect on TE activity (i.e., transposon traps vs reference regions). First, the abundance of different TE families should be more narrowly distributed in transposon traps than in reference regions. Second, the abundance of TEs within and outside of reference regions should be positively correlated, whereas no positive correlation is expected for transposon traps. These differences are robust over a wide range of different scenarios and parameters, such as varying transposition rates, sizes of transposon traps, age of the invasions, negative effects of TEs, and different combinations of these factors. However, we can not fully rule out the possibility that some specific parameter combination or simulation scenario exists where the two key differences do not hold. With simulations, only a finite number of possible scenarios or parameter combinations can reasonably be explored. Nevertheless, our work shows that under the vast majority of the feasible scenarios, for example, a positive correlation between the TE abundance within and outside of transposon traps is not expected.

When simulating the TE composition of reference regions, we assumed that some other region outside of the reference act as transposon traps. It is however possible that TE invasions are not stopped by transposon traps but by an hitherto unknown mechanism (possibly siRNAs; see below [36]). However, even in such a scenario the observed correlation of the TE abundance between reference regions and the rest of the genome will persist. Additionally, the distribution of TE insertion in reference regions will likely become even more heterogeneous than observed in our simulations, where we assumed that some region with the same size as the reference region acts as transposon trap. Therefore, we think that our two key differences between reference regions and transposon traps are conservative.

We examined the observed distribution of piRNA clusters in five *D. melanogaster* strains. We restricted the analyses to these strains because high-quality genome assemblies, genomic reads and small RNA data from ovaries are only available for these strains (small RNA data for Pi2 were generated by us). In principle, a single strain would have been sufficient to test our predictions about the composition of piRNA clusters under the trap model but an analysis of five strains provides a more comprehensive picture, allowing us to rule out that our results are merely based on a strain that may, for example, have assembly problems. Furthermore, we tested the observed TE composition using 3 complementary approaches, each with their own strengths and weaknesses.

Our analysis of the five strains revealed that not a single cluster insertion can be found for several TE families, which is in stark contrast to expectations under the trap model. We can not fully rule out the hypothesis that some insertions in the piRNA clusters were missed, since the assemblies of the five strains may still be incomplete. Only telomere-to-telomere assemblies of the investigated strains, currently available for a single human genome [74], will provide a complete picture of the genomic landscape of *Drosophila*, including its piRNA clusters. However, we consider it unlikely that the missing cluster insertions are a result of insufficient assembly quality. First, apart from the reference genome (Iso-1), the assemblies used in this work are based on long reads, which enable high-quality assemblies even for highly repetitive regions [61, 62, 75, 76]. In agreement with this, multiple quality metrics suggest that the assemblies of the five strains used in this work are of high quality (Additional file 1: Table S1). Furthermore, we found the location of the unique sequences flanking the piRNA clusters of the reference genome in most of our assemblies ( 91.8-97.6%). In comparison with other recently published long-read assemblies [63], our assemblies are among those with the most completely assembled piRNA clusters (Additional file 1: Table S2). Finally, we confirmed that the number of cluster insertions is insufficient for many TE families with an approach that does not rely on the assemblies of the individual strains, but instead is based on short reads aligned to the reference genome. Taken together, we do not think that an insufficient assembly quality can account for the missing cluster insertions. One other possible hypothesis which could explain the missing cluster insertions is that some of the TE families may not yet be silenced by the piRNA pathway. For example, a TE family that is currently spreading in *D. melanogaster* could simply not yet have acquired any insertions in piRNA clusters. Previous genomic scans showed that four TE families invaded *D. melanogaster* during the last century: P-element, I-element, Tirant, and hobo [49]. Since several of our strains were sampled early during the last century, not all of these four TEs are present in our five strains (e.g., the P-element is missing in Iso-1). Therefore, we solely considered TE families that were actually present in a given strain for the analysis of missing cluster insertions (for example we did not consider the P-element in Iso-1). However, Tirant is present in the analyzed Oregon-R assembly [60] but we did not find a cluster insertion. It is thus feasible that Tirant is not yet under host control in this line.

We believe that the most likely explanation for the missing cluster insertions is that TE insertions outside of piRNA clusters could also be producing piRNAs [31]. The conversion of a regular TE insertion into a DSL may be driven by maternally deposited piRNAs [33, 34, 77]. Hence, once the piRNAs targeting an invading TE have emerged, large numbers of TE insertions outside of piRNA clusters may be converted into piRNA producing loci. The DSL generate a substantial redundancy in the number of piRNA producing loci and could thus compensate for the missing cluster insertions. In agreement with this, a recent study demonstrated that the deletion of three major piRNA clusters had no effect on the activity of the resident TE families [46]. The authors suggested that DSL may compensate for the deleted cluster insertions [46]. Based on our genome-wide scan of the five strains, we suggest that about $2-5\%$ of all TE insertions act as DSL (Additional file 1: Fig. S10). When we consider DSL in addition to cluster insertions, we found that most of the TE families, in all five strains, have at least one piRNA producing locus (either DSL or cluster insertion). We therefore conclude that DSL could account for the missing cluster insertions.

However, the DSL cannot account for the over-representation of some TEs in piRNA clusters, nor the correlation between the TE abundance inside and outside of piRNA clusters. Both the over-representation and the correlation of the TE abundance persisted when we merged fragmented TE insertions, only considered full-length insertions or removed piRNA clusters with assembly gaps from the analysis (Additional file 1: Figs. S7–S9). One possible explanation contributing to the over-representation of some families is likely repeat expansion. Hence, some TEs in piRNA clusters may not represent independent insertion events but rather tandem duplications of sub-sequences of the clusters [62]. Both the over-representation of some families and the correlation of the TE abundance are expected for random genomic regions, where a TE insertion has no effect on the activity of the TE. Therefore, it is possible that not all TE insertions in piRNA clusters deactivate a TE. Instead, other mechansims, such as siRNAs generated from dsRNA of TEs, might be responsible for activating the host defence against an invading TE [36]. However, we do not consider it likely that the trap model is entirely incorrect since there is strong evidence that insertions in piRNA clusters can produce piRNAs [41]. Furthermore, insertions in the germline clusters X-TAS and 42AB were shown to silence reporter constructs [36, 40]. Additionally, the transposon ZAM was activated in some strain due to loss of a ZAM insertion in the somatic piRNA cluster *flamenco* and silenced at later generations due to novel insertions in germline piRNA clusters [42, 43].

One possible explanation for the over-representation and the correlation is that the number of cluster insertions required for silencing a TE varies among the TE families. It could, for example, be speculated that silencing of short TEs requires more cluster insertions than silencing of long TEs, since short TEs may generate fewer piRNAs. However, so far no evidence exists for such heterogeneity between the TE families.

Another potential alternative explanation for both the over-representation and the correlation is that not the entire sequence of the piRNA clusters acts as random region (where insertions have no effect on TE activity) but rather only certain regions within the clusters. Hence, TE insertions in some clusters may activate the host defence against an invading TE while insertions in other clusters may have no effect. It is even feasible that this silencing capacity varies within a cluster. In agreement with this, previous studies found that the number of TE insertions in X-TAS (a piRNA cluster) necessary to silence a TE varies among strains: in one strain a single insertion was sufficient, while in another strain two insertions were required [78, 79]. This heterogeneity among the strains may be due to different insertion sites of the TE in X-TAS. To test the hypothesis that the silencing capacity varies among (within) clusters, it would be important to insert artificial sequences into many regions of different piRNA clusters and test if these insertions repress a reporter (e.g., similarly to Luo et al. [36] and Josse et al. [40]). In agreement with this hypothesis, a recent study using transgenes revealed such a heterogeneity within X-TAS [80].

The over-representation and the correlation could also be due to a rapid turnover of the location of piRNA clusters. It is thought that the position of piRNA clusters in the next generation is not determined at the genomic level, e.g., due to sequence motifs, but rather by maternally deposited piRNAs. It is not clear how stably the piRNA composition is inherited over many generations but it is feasible that such epigenetic transmitted information may be subject to some variation over the course of time. In agreement with this, recent studies found a rapid turnover in the location and composition of piRNA clusters [30, 46]. This raises the possibility that some of the piRNA clusters were solely established after an invading TE was silenced by the host. For TEs that have invaded before a cluster was established, the region of the soon to be established cluster would have acted much like a random region with no effect on TE activity.

In summary, we think that the heterogeneity of piRNA clusters both temporally (rapid turnover of the location) and spatially (varying silencing capacity within cluster) is likely responsible for both the observed over-representation of some TE families in piRNA clusters and the correlation of the TE abundance within and outside of piRNA clusters. It may be a promising avenue of future work to further investigate this heterogeneity of the clusters.

Our work also raises the important open question as to which role piRNA clusters play in stopping TE activity. It is feasible that piRNA clusters are important for activating the piRNA-based host defence but once the host defence is established, piRNA clusters may become less important due to the redundancy of piRNA producing loci for example generated by multiple DSL [81]. It is also possible that the silencing of an invading TE is not triggered by insertions into piRNA clusters but rather by siRNAs [36]. In this scenario, insertions in piRNA clusters may not be necessary to trigger the piRNA-based host defence against an invading TE. Replicated TE invasions in strains with a defective siRNA pathway and strains lacking major piRNA clusters may be a promising approach to address these open questions. These hypothesis are not mutually exclusive. It is thus feasible that silencing of a TE invasion can be triggered by an insertion into a piRNA cluster or may emerge de novo, e.g., mediated by siRNAs. In line with this, a recent study found evidence for both the trap model as well as the de novo model [82].

## Conclusions

In this work, we investigated the trap model, i.e., the notion that an invading TE is silenced by insertions in piRNA clusters, from a population genetics perspective. We found that the composition of piRNA clusters in five high-quality assemblies of different *D. melanogaster* strains is not in agreement with expectations under the simple trap model. Dispersed piRNA producing TE insertions and temporal as well as spatial heterogeneity of piRNA clusters may account for these deviations.

## Methods

### Data of fly strains

In this work we analyzed the five *D. melanogaster* strains Canton-S, DGRRP-732, Iso-1, Oregon-R, and Pi2.

As a part of our previous work, we generated high-quality assemblies for Canton-S (GCA_015832445.1) and Pi2 (GCA_015852585.1) [62, 83, 84]. We also used the assemblies of the *D. melanogaster* reference strain Iso-1 (GCA_000001215.4 [59, 85], DGRP-732(GCA_004798075.2) [61, 86] and Oregon-R (GCA_003402015.1) [60, 87]. Genomic short-read data for these strains have been made available (SRX8038113, SRX8038116, SRX8038119, SRX006167, SRX671607) [57, 58, 62, 88–92]. We previously published the small RNA data from ovaries of Canton-S (SRX8396898) and Iso-1 (SRX8396899) [49, 93, 94]. We also used the ovarian small RNA data of DGRP-732 (SRX698089) [65, 95] and Oregon-R (SRX22795339) [66, 96].

### Small RNA sequencing

The Pi2 strain was obtained from Bloomington Drosophila Stock Center (RRID:BDSC_2384), raised on standard food at $25^\circ C$. To obtain small RNA data for Pi2 we extracted total RNA from ovaries using TRIzol (Invitrogen, Carlsbad, CA). The small RNA library preparation and sequencing was performed by Fasteris (Geneva, Switzerland). RNAs were separated in a polyacrylamide gel electrophoresis and the abundant 2S rRNA was depleted. The libraries were prepared using the the Illumina TruSeq small RNA kit and sequenced on the Illumina HiSeq systems.

### Simulations

TE invasions were simulated using our tool “Invade” (v0.808, [20]). For this work, we added a novel feature which allowed us to specify the position of reference regions (i.e., genomic regions with no effect on TE activity).

Similar to our previous work [21], we simulated diploid organism with 5 chromosomes, each 10Mb in size. We used a uniform recombination rate of 4cM/Mb. Transposons traps (piRNA clusters) and reference regions, each accounting for 3.5% of the genome, were simulated on opposite ends of the chromosomes. We assumed that a TE insertion in a transposon trap silences the TE while an insertion into a reference region has no effect. We simulated populations of 1, 000 diploid individuals using non-overlapping generations for 10, 000 generations. To avoid the early stochastic phases of TE invasions, where TEs are frequently lost due to genetic drift [47], we triggered each invasion by randomly distributing 1, 000 TE insertions in the population (population frequency ${f= 1/(2\cdot 1000)}$). Unless mentioned otherwise, we used a constant transposition rate of $u=0.1$. Individuals with a TE insertion in a piRNA cluster had a transposition rate of $u=0.0$. Simulations with negative selection were performed using the fitness function $w=1-\sum _{{i=1}}^{n}{x_i}$ where *w* is the fitness of an individual and $x_i$ the negative effect of each TE insertion. We terminated a simulation when the average fitness fell below $<0.1$ (extinction of the population).

Since Invade reports TE insertions per diploid individuals, TE counts were divided by 2.0 to obtain estimates for haploid genomes.

### Identification of TEs

For the identification of TEs, we used the consensus sequences of TEs in *D. melanogaster* and added the sequences of Chimpo, Chouto, Pifo, Batumi, and Bica [97] (see data availability). To detect TE insertions based on short-read data, we relied on our tool PopoolationTE2 (v1.10.04) [69]. We use the release 5 of *D. melanogaster* reference genome, as a widely used standard annotation of piRNA clusters is available for this release [24]. In agreement with the manual of PoPoolationTE2, we first built a FASTA-file consisting of the repeat-masked (RepeatMasker version 4.0.7, [70]) reference genome and the consensus sequences of TEs. We mapped the reads to this FASTA-file using bwa mem (version 0.7.17-r1188) and the option -M (mark secondary alignments [98]. We generated a ppileup file (–map-qual 15), identified signatures of TE insertions (–min-count 2 –signature-window minimumSampleMedian), estimated population frequencies, and paired-up signatures of TE insertions. To exclude unreliable and somatic insertions, we solely considered TEs with a minimum population frequency of 0.3. TE insertions with frequencies lower than 0.6 were assumed to be heterozygous. To obtain the number of TE insertions per haploid genome, the abundance of heterozygous insertions were divided by two (the number of homozygous insertions $\ge 0.6$ were not altered). We used RepeatMasker (4.0.7) to identify TE insertions in the assemblies of the five strains (-s -no_is -nolow [70]). To prevent fragmented TE annotations, we set the $--frag$ option to 40, 000, 000, which is higher than the largest scaffold in our data.

We filtered for TE insertions with a minimum length of 100bp and a maximum divergence of 10% from the consensus sequence. Finally, we excluded families that were not recently active ($\ge 25\%$ population frequency [54] and families which are only active in the soma (as these TEs are controlled by a distinct piRNA cluster [29]). The TE families considered in this work are shown in the Additional file 1: Table S5.

### Annotation of piRNA clusters

We used our CUSCO approach [62] to lift-over the classic annotations of piRNA clusters to the assemblies of the five strains. We identified sequences flanking the piRNA clusters in (release 5 [24]) and aligned these sequences to the five assemblies using bwa sw(version 0.7.17-r1188, [98]). The regions between these two aligned sequences were annotated as piRNA clusters. Telomeric associated sequences (TAS) frequently act as piRNA clusters [40] but these clusters are not flanked by a unique pair of sequences. To identify these clusters, we aligned the most distal gene of each chromosome arm (release 6, [59]) to the assemblies of the five strains and annotated the region between this gene and the end of the contig as TAS cluster. Overlapping piRNA clusters were merged using bedtools (v2.27.1, [99]).

We used proTRAC (V.2.4.4, [100]) and ovarian small RNA data to de novo annotate piRNA clusters in the five assemblies. We trimmed reads using cutadapt [101], filtered reads with a length of 23-29nt, and mapped the reads to a set of *D. melanogaster* mRNAs, miRNAs, rRNAs, snRNAs, snoRNAs, tRNAs, and TEs [97, 102] using NovoAlign (V3.09.00, [103]). Based on these alignments, we removed reads mapping to a miRNA, mRNA, rRNA, snRNA, tRNA, and snoRNA. Following the proTRAC pipeline, we collapsed overlapping reads, removed low complexity reads, aligned the remaining reads to the assemblies of the five strains, and run proTRAC using uniquely mapping reads (-pdens 0.05 -pimin 23 -pimax 29 -1Tor10A 0.3 -clsize 5000 -clstrand 0.5) [100]. Following Gebert et al. [46], neighboring clusters were joined if the distance between clusters was smaller than their combined lengths.

### Quality of the assembies

We ran BUSCO (v5.0.0) for all assemblies using the augustus mode and the *diptera_odb10* data set [104]. CUSCO values (the fraction of completely assembled piRNA clusters) were computed based on alignments of unique sequences flanking piRNA clusters (see above) in the reference assembly [62] using bwasw (0.7.17-r1188 [98]). We delineated between gapped (g.CUSCO) and ungapped CUSCO (u.CUSCO) which only considers piRNA clusters without any assembly gaps. To calculate coverage quality and softclip quality values for clusters [62], we mapped long reads of the corresponding strain to the assemblies using minimap2 (v2.16-r922 [105])[60–62, 106–111].

Only reads with a minimum mapping quality of 60 and minimum read length of 5 kb were considered. Assembly sizes and N50 values were obtained from the FASTA index files generated by samtools (v1.9 [112]).

### DSL detection

For finding the asymmetric piRNA signatures of DSL, we solely considered reads with a length between 23 and 29 nt. We furthermore excluded reads mapping to miRNAs, rRNAs, snRNAs, snoRNAs, and tRNAs. The remaining reads were mapped to the corresponding assembly using NovoAlign (V3.09.00, [103]). DSL were identified based on uniquely mapping reads (minimum mapping quality 5). To estimate the rate of false positive DSL, we considered BUSCO genes with a minimum length of 100 bp. For each feature (TE or BUSCO gene), we estimated the number reads aligning 500 bp downstream or upstream of the insertion. As DSL we only considered insertions with a minimum of 5 antisense reads per million in the upstream region and 5 sense reads per million in the downstream region. TE insertions in piRNA clusters were not considered as DSL.

### Ping-pong signatures

Ping-pong signatures were computed based on small RNAs mapped to the *D. melanogaster* TE consensus sequences (see above) using the script https://sourceforge.net/p/te-tools/code/HEAD/tree/piRNA/ping-pong-signature.py (last access June 19, 2023) and reads mapping with up to two mismatches. The *Z*-scores were computed as described previously [45].

### Supplementary information

**Additional file 1:** **S1-S13**, **Table S1-S5.** **Figure S1.** Transposon trap sizes. **Figure S2.** Strength of negative selection. **Figure S3.** Strength of negative selection including trap insertions. **Figure S4.** Number of loci under negative selection. **Figure S5.** Random strengths of negative selection. **Figure S6.** Correlation between the TE abundance in piRNA clusters and the rest of the genome for individual genotypes. **Figure S7.** Analysis with with merged TE fragments. **Figure S8.** Analysis with full-length copies. **Figure S9.** Analysis of ungapped clusters. **Figure S10.** Heterogeneity of DSL abundance. **Figure S11.** Distribution of piRNA source loci. **Figure S12.** Abundance of piRNA producing loci. **Figure S13.** Distribution of ping-pong Z-scores. **Table S1.** General assembly statistics. **Table S2.** CUSCO of different genome assemblies. **Table S3.** TE families without piRNA cluster insertions. **Table S4.** Z-scores of ping-pong signatures. **Table S5.** TE families considered for analyses in this work.

## Data Availability

The ovarian small RNA data of the *D. melanogaster* strain Pi2 have been deposited in the NCBI BioProject database under the accession number PRJNA930650 [113]. The new version of Invade is available at SourceForge (https://sourceforge.net/projects/invade/) [114] (last access on September 15, 2023). All analyses performed in this work were documented with R Markdown and made available at GitHub (https://github.com/filwierz/trapmodel) [115] (last access on September 15, 2023). The used scripts and the TE library are also available at this repository.

## Abbreviations

DSL: Dispersed source loci
g.CUSCO: Gapped CUSCO
TAS: Telomeric associated sequences
TE: Transposable element
TSC: Transposition, selection, piRNA clusters
u.CUSCO: Ungapped CUSCO
