# Supplementary material for: The composition of piRNA clusters in Drosophila melanogaster deviates from expectations under the trap model
Source: BMC Biol. 2023 Oct 20;21:224. doi: 10.1186/s12915-023-01727-7 (PMC10588112; doi:10.1186/s12915-023-01727-7)
Supplement: Supplementary file 1 — Additional file 1: S1-S13, Table S1-S5. Figure S1. Transposon trap sizes. Figure S2. Strength of negative selection. Figure S3. Strength of negative selection including trap insertions. Figure S4. Number of loci under negative selection. Figure S5. Random strengths of negative selection. Figure S6. Correlation between the TE abundance in piRNA clusters and the rest of the genome for individual genotypes. Figure S7. Analysis with with merged TE fragments. Figure S8. Analysis with full-length copies. Figure S9. Analysis of ungapped clusters. Figure S10. Heterogeneity of DSL abundance. Figure S11. Distribution of piRNA source loci. Figure S12. Abundance of piRNA producing loci. Figure S13. Distribution of ping-pong Z-scores. Table S1. General assembly statistics. Table S2. CUSCO of different genome assemblies. Table S3. TE families without piRNA cluster insertions. Table S4. Z-scores of ping-pong signatures. Table S5. TE families considered for analyses in this work. [file 12915_2023_1727_MOESM1_ESM.pdf]

Additional file 1:
Supplementary figures and tables

Filip Wierzbicki[1,2], Robert Kofler[1,*]

[1]Institut für Populationsgenetik, Vetmeduni Vienna, Vienna, Austria
[2]Vienna Graduate School of Population Genetics, Vienna, Austria

List of Figures

S1 Transposon trap sizes . . . 2
S2 Strength of negative selection . . . 3
S3 Strength of negative selection including trap insertions . . . 4
S4 Number of loci under negative selection . . . 5
S5 Random strengths of negative selection . . . 6
S6 Correlation between the TE abundance in piRNA clusters and the rest of the genome for individual genotypes 7
S7 Analysis with with merged TE fragments . . . 8
S8 Analysis with full-length copies . . . 9
S9 Analysis of ungapped clusters . . . 10
S10 Heterogeneity of DSL abundance . . . 11
S11 Distribution of piRNA source loci . . . 12
S12 Abundance of piRNA producing loci . . . 13
S13 Distribution of ping-pong Z-scores . . . 14

List of Tables

S1 General assembly statistics . . . 15
S2 CUSCO of different genome assemblies . . . 16
S3 TE families without piRNA cluster insertions . . . 17
S4 Z-scores of ping-pong signatures . . . 18
S5 TE families considered for analyses in this work . . . 19

Supplementary figures

*correspondence to rokofler@gmail.com

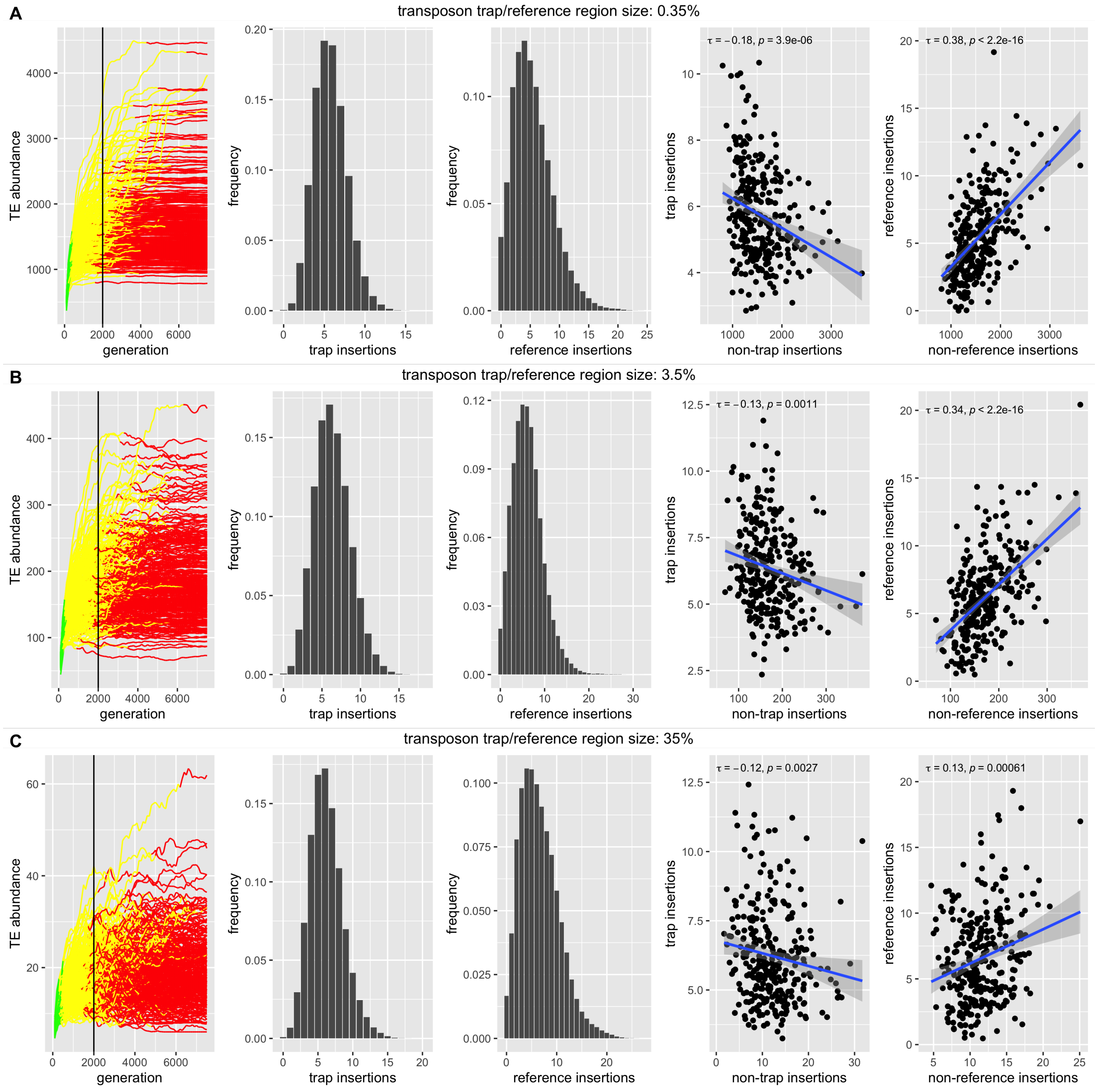

Figure S1: Effect of the size of transposon trap and reference regions on our two key metrics (the distribution and correlation of TEs in trap and reference regions). Neutral simulations with transposon trap and reference region size of A) 0.35% B) 3.5% and C) 35% of the genome. The transposition rate was $u = 0.1$.

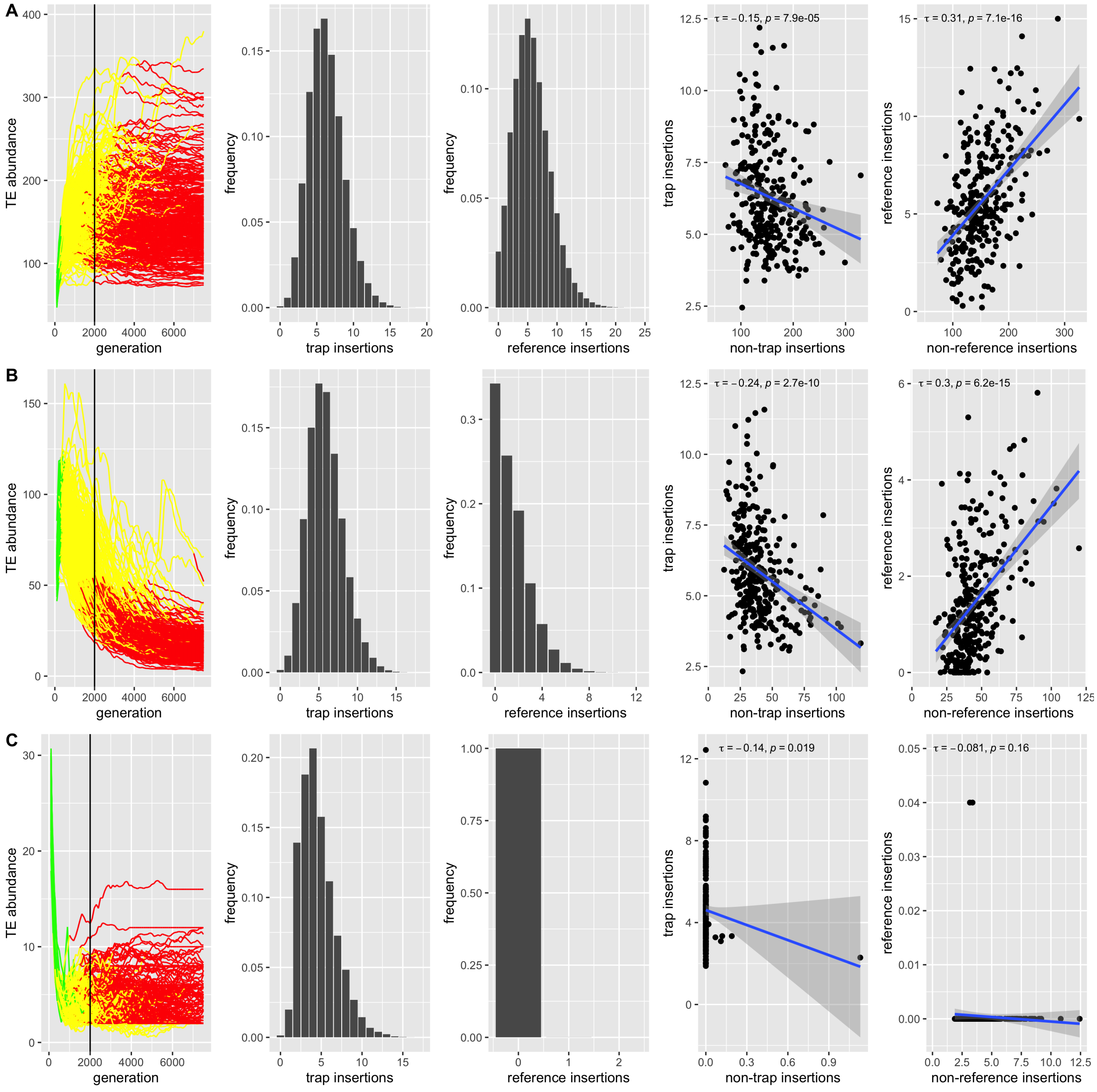

Figure S2: Effect of negative selection, excluding trap insertions, on our two key metrics. Except for TE insertions in transposon traps, each TE insertion reduces the fitness of the host by A) 0.0001 B) 0.001 and C) 0.01. The transposition rate was $u = 0.1$

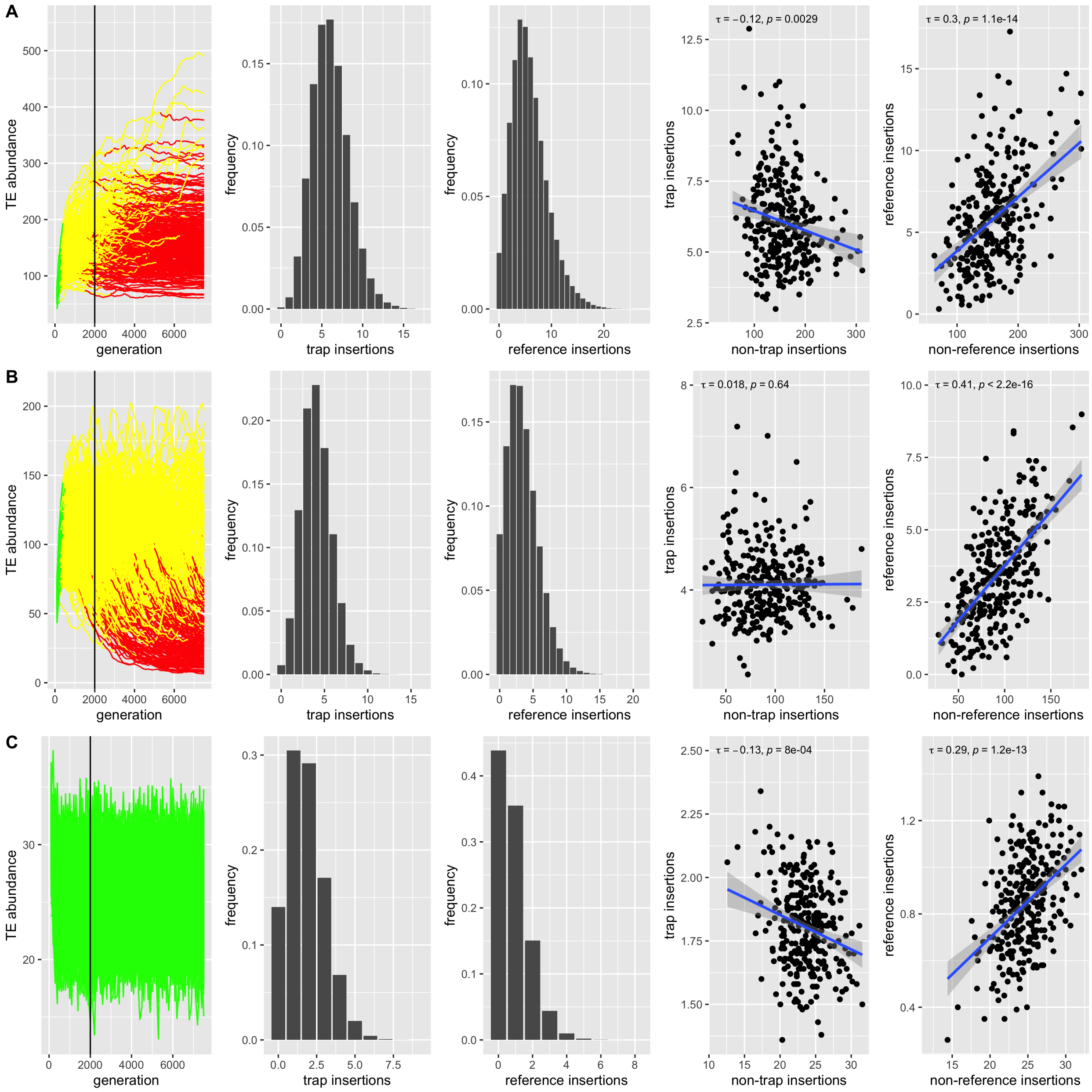

Figure S3: Effect of negative selection, including trap insertion, on our two key metrics. Each TE reduces the fitness of the host by A) 0.0001 B) 0.001 and C) 0.01. The transposition rate was $u = 0.1$.

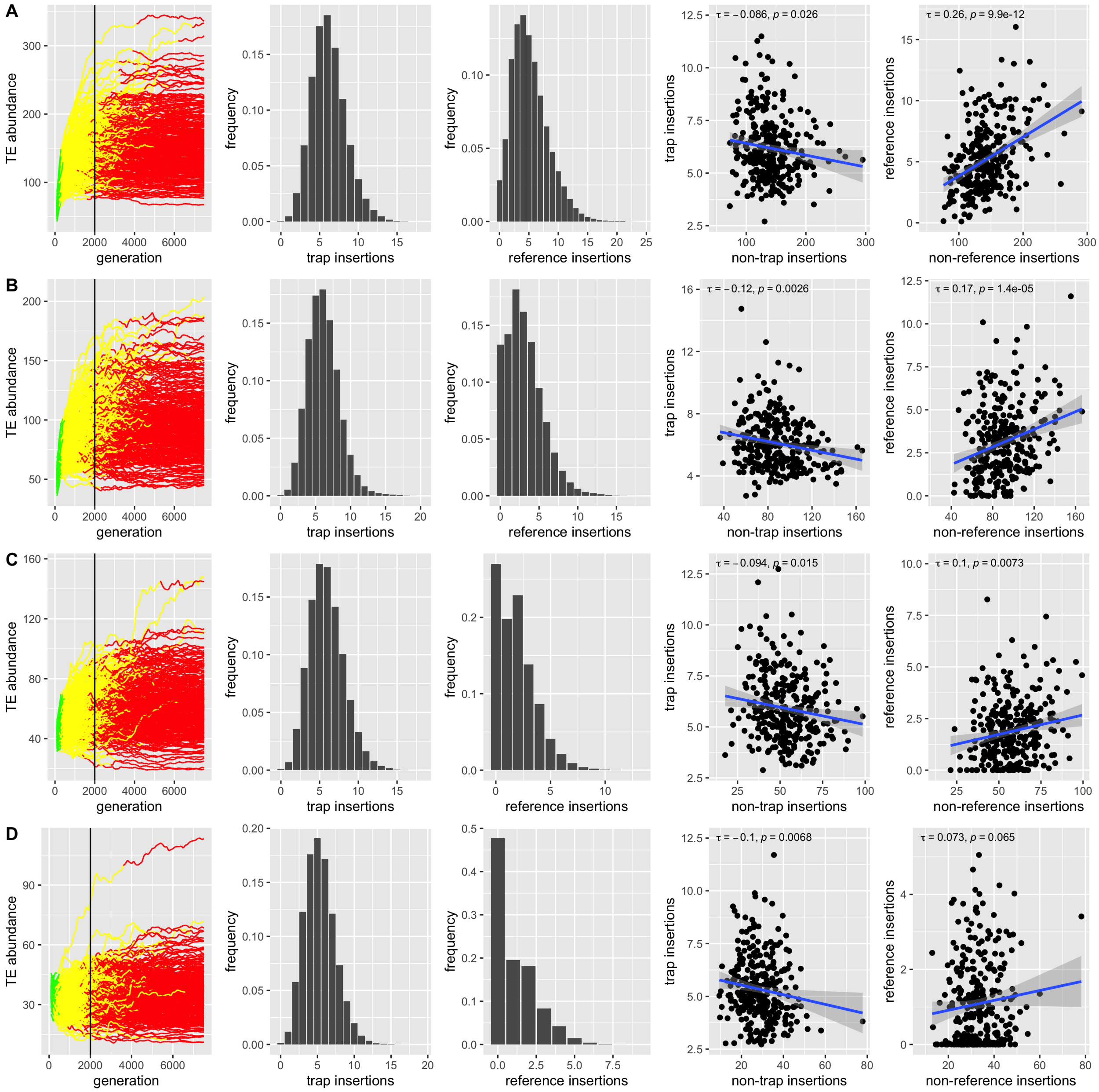

Figure S4: Effect of the fraction of negatively selected TEs on our two key metrics. Except for transposon traps, the following fraction of TE insertions had a negative effect ($x = 0.01$) on host fitness host A) 10% B) 30% C) 50% and D) 70% of the genome. The remaining TE insertions (e.g. for A: $100 - 10 = 90\%$) were neutral ($x = 0.0$). We used a transposition rate of $u = 0.1$ and site-specific negative effects of TEs (hence independent TE insertions at the same site will always have the same fitness effect).

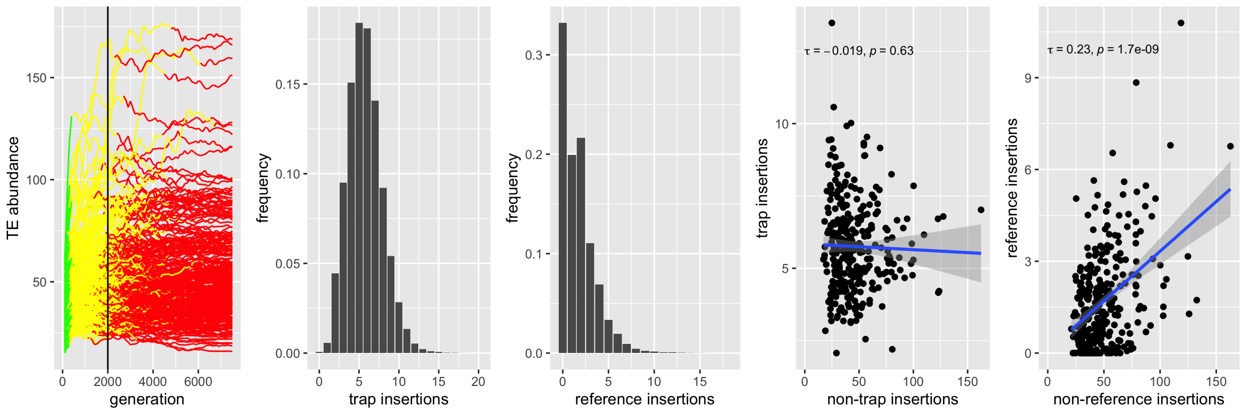

Figure S5: Effect of varying the deleterious effect of TE insertions among replicates on our two key metrics. Each replicate may be regarded as the invasion of a different TE family with distinct fitness effects to hosts. For each of the 300 replicates, we randomly picked a negative effect of TEs between $x = 0.001$ and $x = 0.1$ for 40% of the genome while TE insertions into the remaining 60% were neutral. The transposition rate was $u = 0.1$.

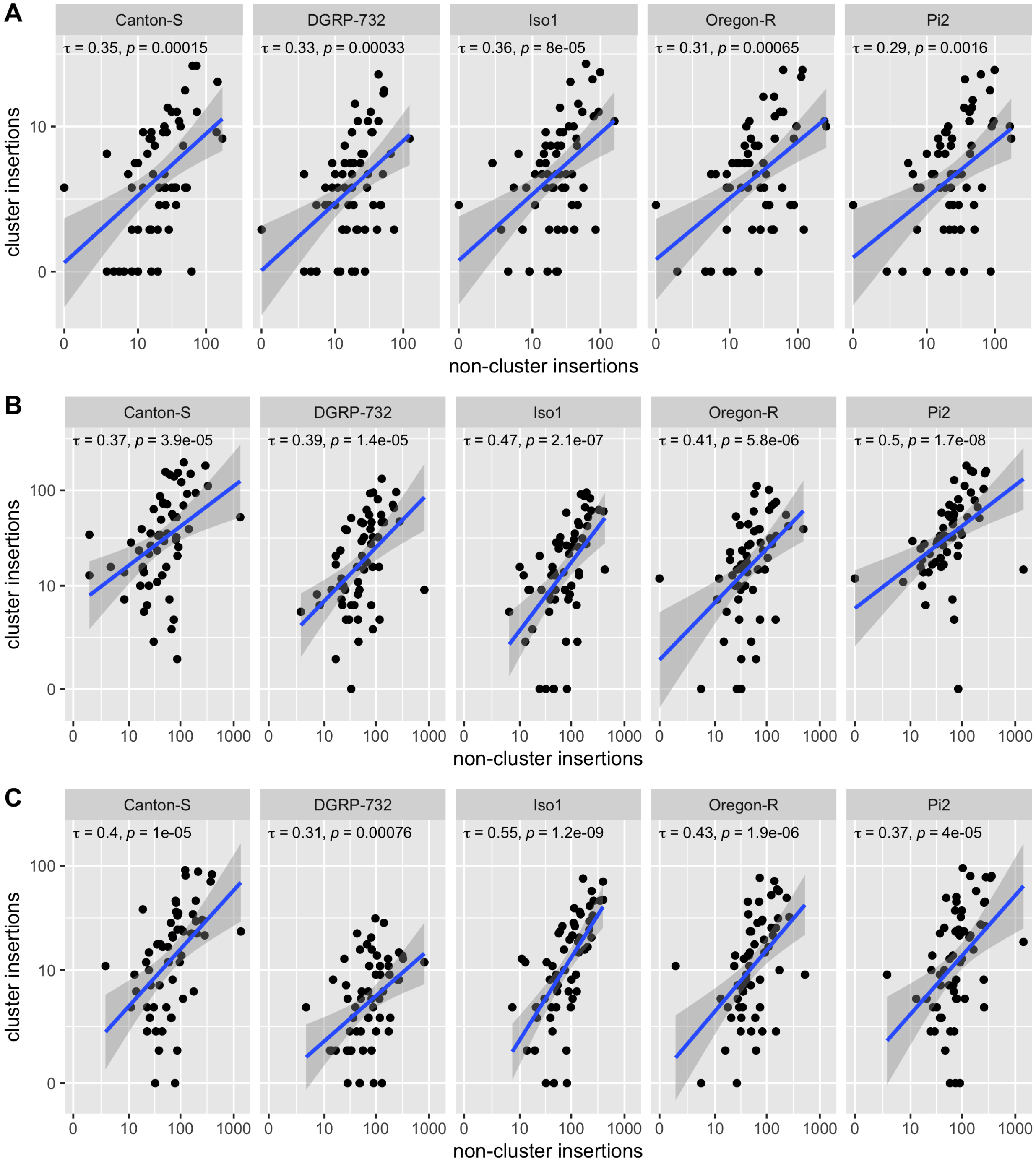

Figure S6: Correlation between the number of TE insertions in piRNA clusters and the rest of the genome (non-cluster insertions). The TE abundance in these two regions was estimated with three different approaches. A) Short reads aligned to the reference genome with the annotations of piRNA clusters. B) Strain-specific assemblies - annotations of piRNA clusters were lifted-over to the assemblies based on unique sequences flanking the clusters. C) Strain-specific assemblies - *de novo* annotations of piRNA clusters. All counts are copy numbers per haploid genome and Kendall's rank correlations were used.

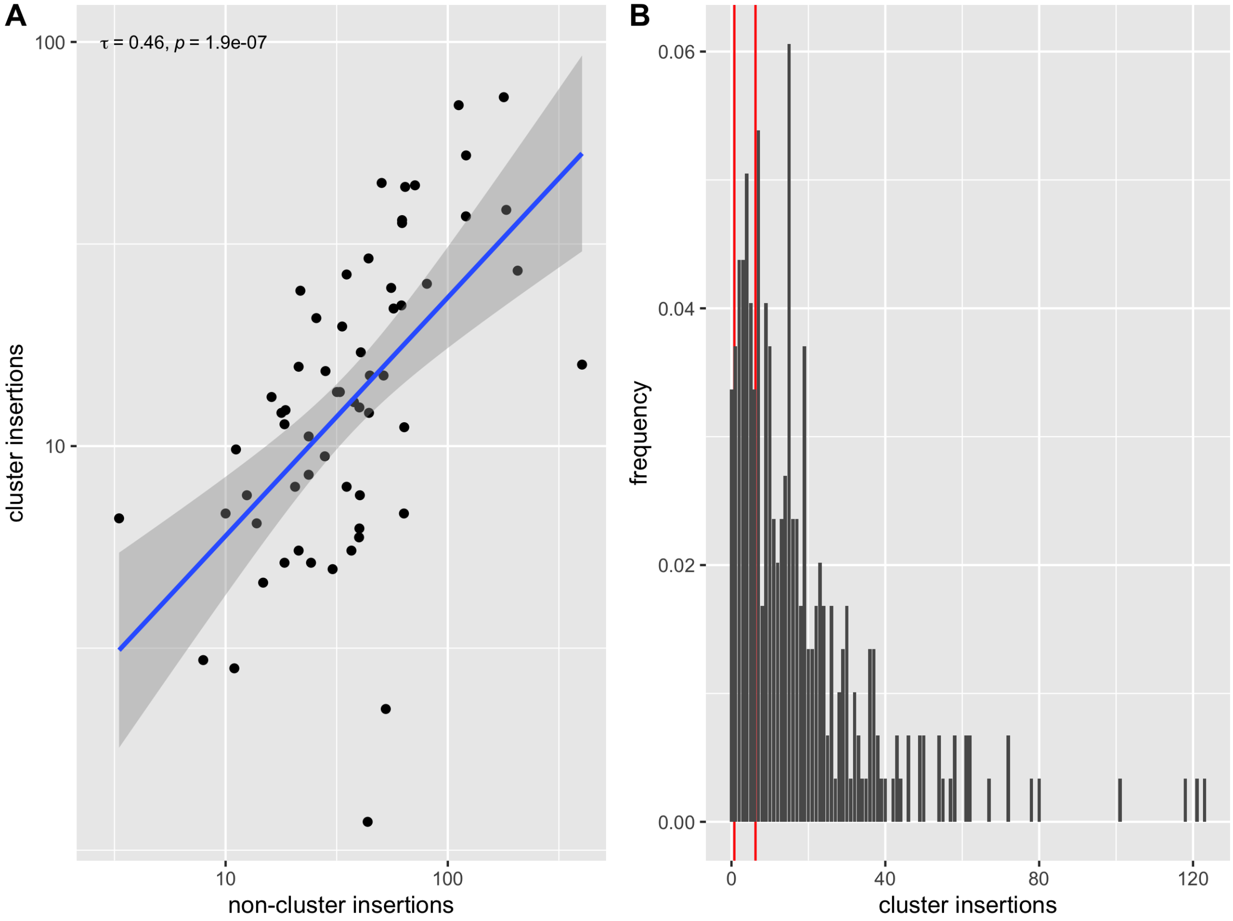

Figure S7: Effect of merging fragmented TEs on the correlation between cluster and non-cluster insertions (A) and the distribution of TE insertions in piRNA clusters (B). Copy numbers are average number of insertions per TE family and haploid genome.

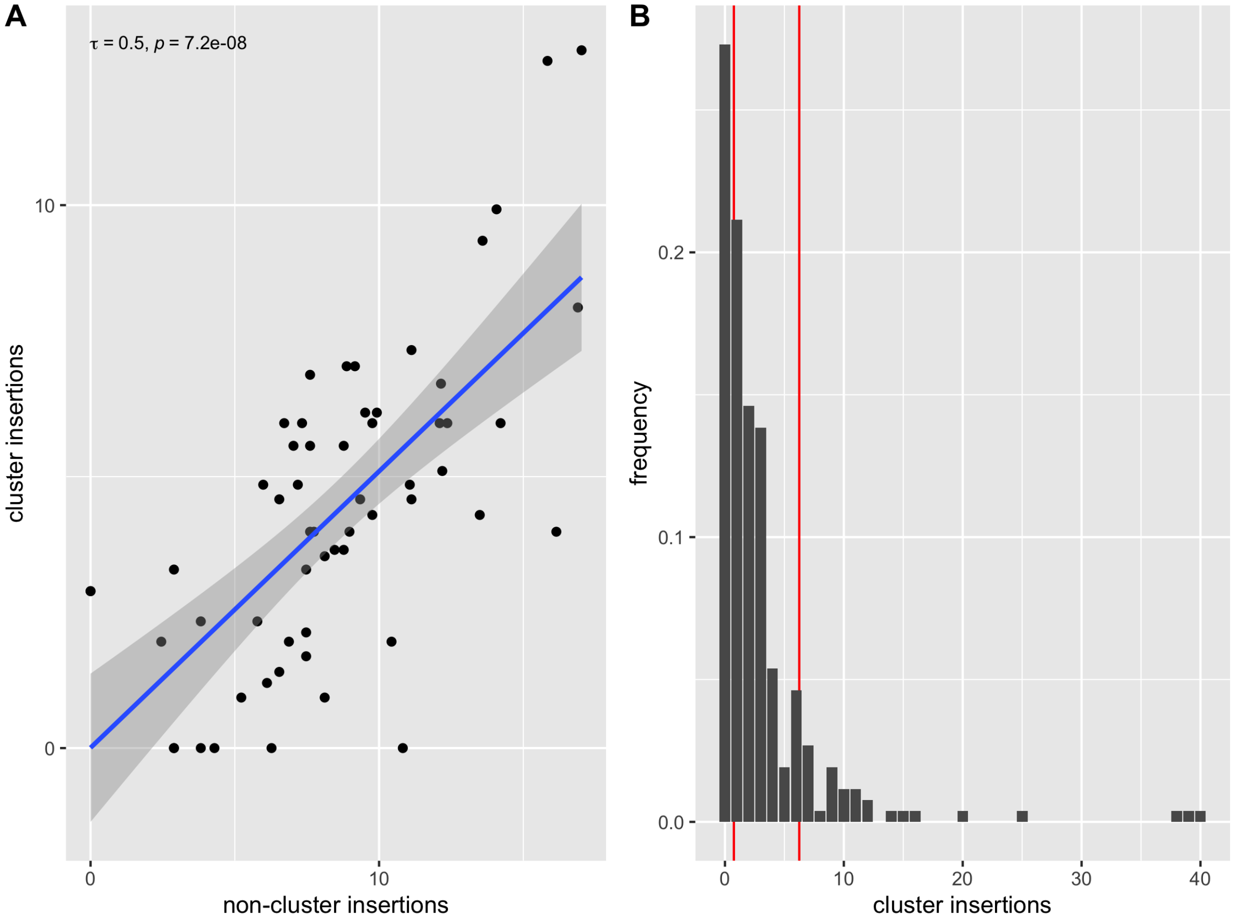

Figure S8: Correlation between cluster and non-cluster insertions (A) and the distribution of TE insertions in piRNA clusters (B) when solely full-length insertions are considered. Copy numbers are average number of insertions per TE family and haploid genome.

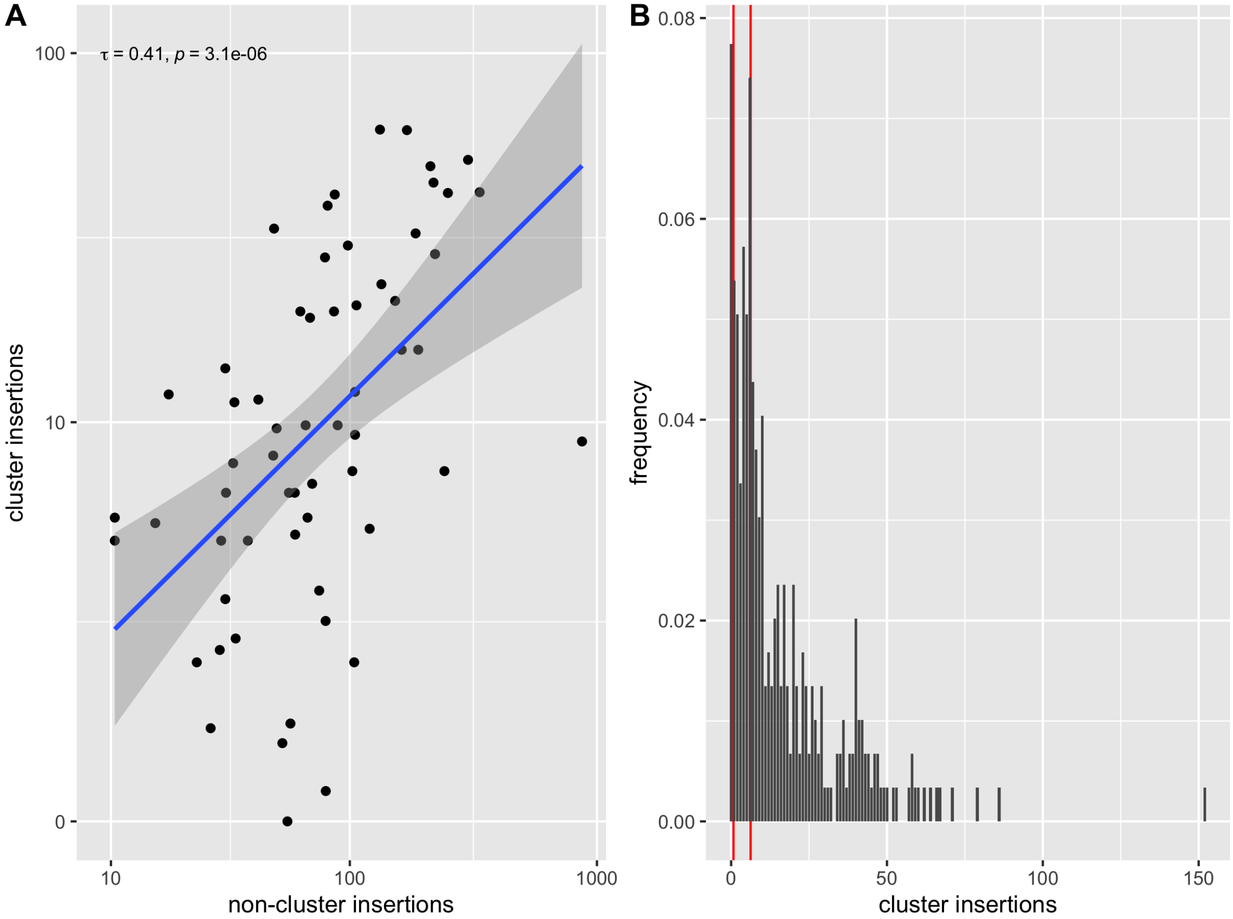

Figure S9: Correlation between cluster and non-cluster insertions (A) and the distribution of TE insertions in piRNA clusters (B) when solely completely assembled piRNA clusters (without assembly gap) are considered.

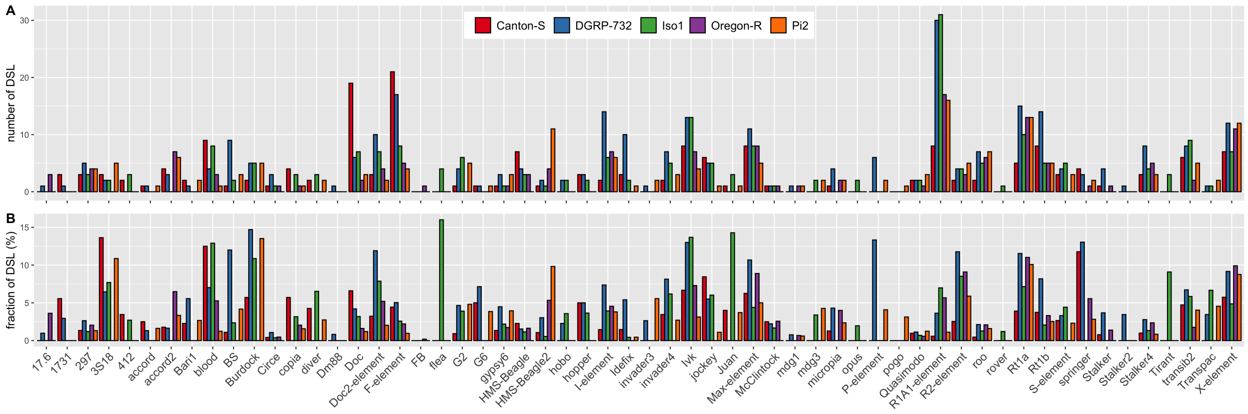

Figure S10: Heterogeneity of the abundance of DSL among TE families and fly strains. A) Absolute numbers of DSL for each TE family in the five strains. B) Fracton of DSL for each TE family in the five strains.

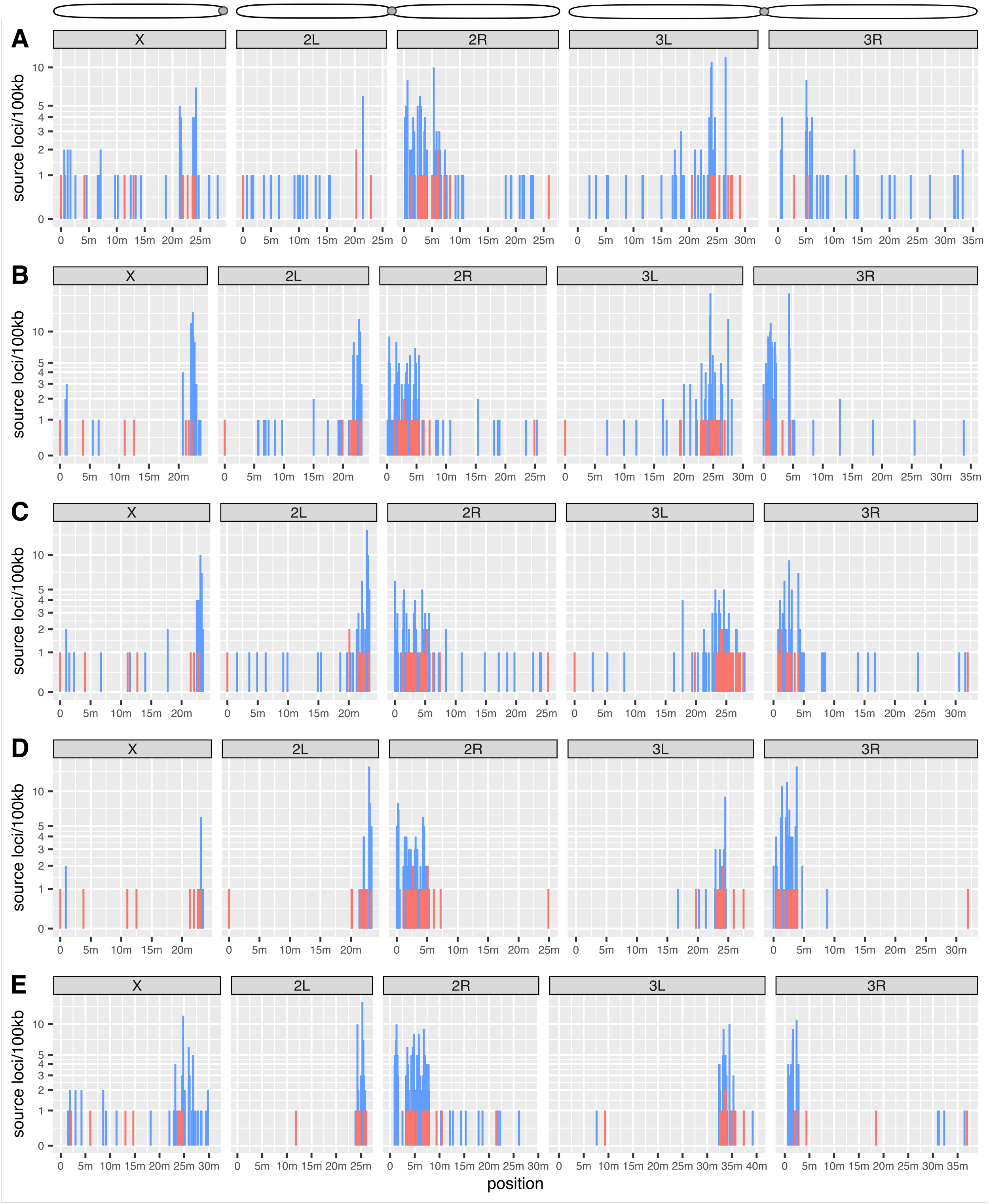

Figure S11: Distribution of piRNA source loci in the five strains (A-E). The abundance in 100kb windows is shown for TE insertions in piRNA clusters (red) and DSL (blue). The reverse complement is shown for chromosome arm 2L of Pi2 (the strand of the published assembly is likely not identical to the strand of the reference genome). A: Canton-S, B: DGRP-732, C: Iso1, D: Oregon-R, E: Pi2. A cartoon of chromosomes with gray circles corresponding to centromeres is shown above.

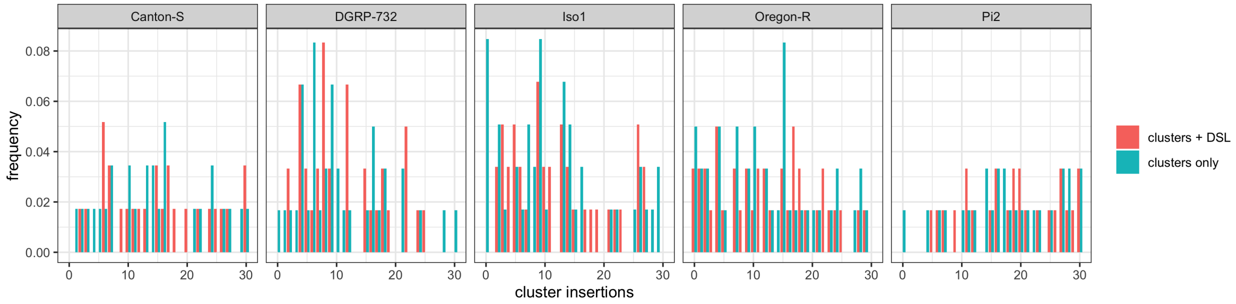

Figure S12: Abundance of piRNA producing loci in the five strains. Data are shown for cluster insertions (red) and cluster insertions plus DSL (cyan). Note that the number of families without piRNA producing locus is dramatically reduced when DSL are considered.

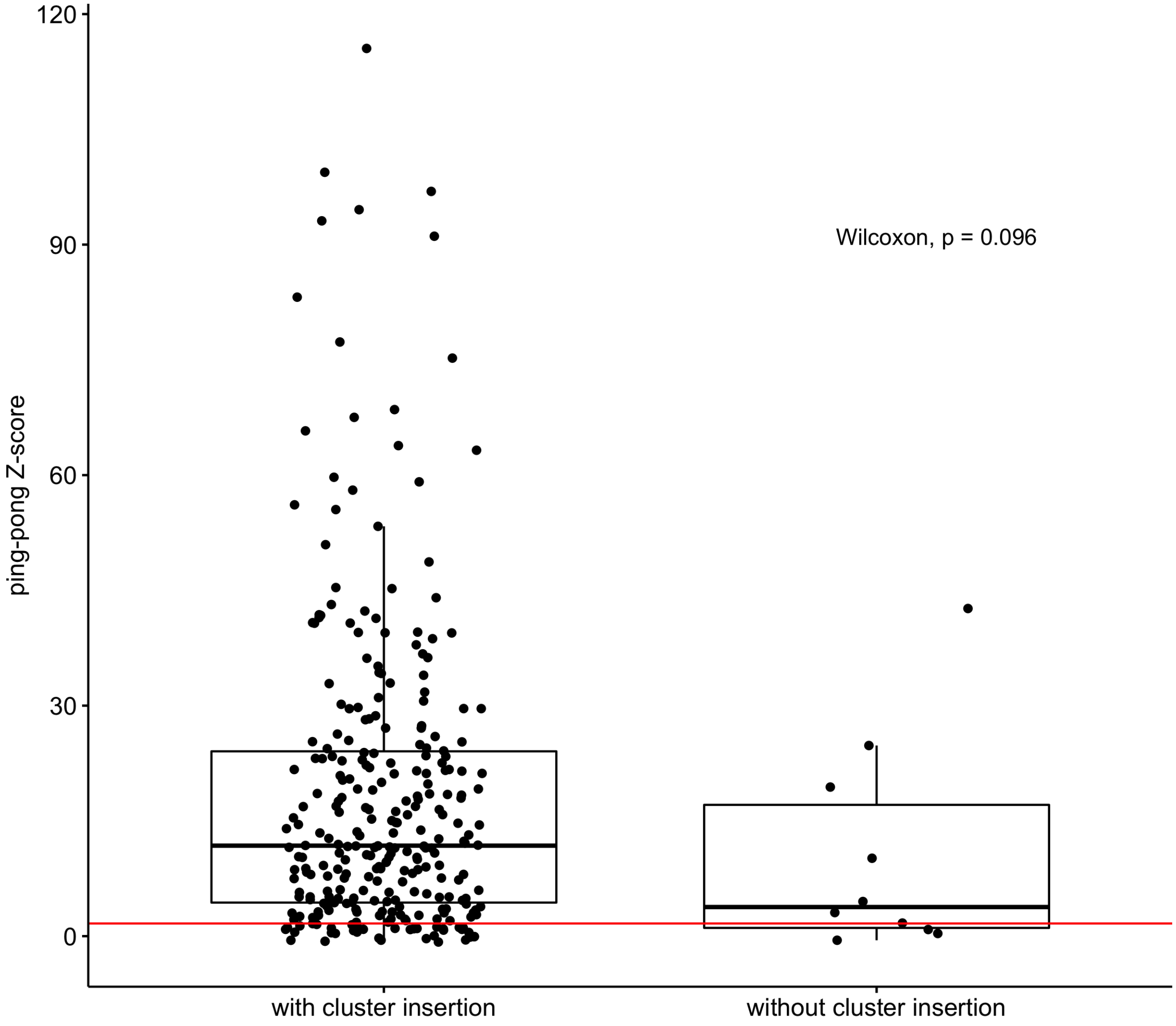

Figure S13: Distribution of Z-scores of ping-pong signatures for all investigated TEs and strains. A Z-score of 1.65 (red line) or higher corresponds to a significant ping-pong signature ($p = 0.05$). The distributions of Z-scores are similar for TE families with and without insertion in a piRNA cluster (Wilcoxon rank sum test, $W = 1874$, $p = 0.096$)

Table S1: General assembly statistics

Assembly	Canton-S	DGRP-732	Iso1	Oregon-R	Pi2
Assembly size (MB)	149.1	141.6	143.7	136.3	167.8
N50 (MB)	28.2	25.7	25.3	25.1	30.9
BUSCO score (%)	99.4	99.0	99.5	99.2	99.3
g.CUSCO (%)	95.3	92.9	97.6	91.8	97.6
u.CUSCO (%)	78.8	75.3	85.9	76.5	81.2
Coverage quality-all	0.069	0.107	0.141	0.139	0.066
Coverage quality-ungapped	0.141	0.120	0.192	0.194	0.099
Softclip quality-all	0.512	0.145	0.099	0.329	0.351
Softclip quality-ungapped	0.395	0.162	0.193	0.317	0.282

Table S2: CUSCO of different genome assemblies. Assemblies are sorted by the u.CUSCO. Note the assemblies used in this work (bold) are among those with the highest CUSCO values.

assembly	u.CUSCO (%)	g.CUSCO (%)
**Iso1**	**85.88**	**97.65**
GCA_020141655	84.71	97.65
GCA_020142105	83.53	96.47
GCA_020141675	82.35	96.47
**Pi2**	**81.18**	**97.65**
GCA_020141955	80.00	96.47
GCA_020141575	78.82	96.47
**Canton-S**	**78.82**	**95.29**
GCA_020142005	77.65	94.12
GCA_020141765	77.65	92.94
**Oregon-R**	**76.47**	**91.76**
GCA_020141925	76.47	96.47
**DGRP-732**	**75.29**	**92.94**
GCA_020169495	72.94	92.94
GCA_020142085	72.94	96.47
GCA_020141495	71.76	96.47
GCA_020141935	70.59	98.82
GCA_020141835	70.59	95.29
GCA_020141845	69.41	92.94
GCA_020141585	69.41	95.29
GCA_020141795	65.88	92.94
GCA_020141875	63.53	96.47
GCA_020141515	62.35	85.88
GCA_020141505	61.18	94.12
GCA_020141595	58.82	97.65
GCA_020141705	57.65	92.94
GCA_020141485	57.65	92.94
GCA_020142025	56.47	92.94
GCA_020142045	55.29	96.47
GCA_020141855	52.94	96.47
GCA_020141745	51.76	94.12
GCA_020141625	51.76	90.59
GCA_020141815	49.41	90.59
GCA_020141735	48.24	82.35
GCA_020141985	45.88	87.06
GCA_020141665	43.53	88.24
GCA_020142055	36.47	92.94

Table S3: TE families without piRNA cluster insertions

family	non-cluster	DSL	strain
R2-element	34	4	DGRP-732
diver	46	3	Iso1
R2-element	47	4	Iso1
flea	25	4	Iso1
jockey	83	5	Iso1
Tirant	33	3	Iso1
Bari1	27	0	Oregon-R
Tirant	5	0	Oregon-R
R2-element	33	3	Oregon-R
R2-element	85	5	Pi2

Table S4: Z-scores of ping-pong signatures for all investigated TE families in the five assemblies. A Z-score of 1.65 (red line) or higher corresponds to a significant ping-pong signature ($p = 0.05$). "NA" indicates TE families not having an insertion of the given family and Z-scores of families not having a single cluster insertions are shown in bold.

TE	Canton-S	DGRP-732	Iso1	Oregon-R	Pi2
17.6	12.11	23.49	4.91	59.72	16.47
1731	45.23	19.02	29.62	29.76	13.99
297	16.72	11.45	21.47	0.88	7.74
3S18	16.13	41.83	8.79	11.67	8.03
412	13.08	7.08	14.7	10.49	1.13
accord	5.51	40.74	8.52	4.76	16.89
accord2	0.96	22.26	1.53	3.55	0.35
Bari1	0.86	22.95	1	**4.51**	1.07
blood	41.46	3.51	48.7	24.11	20.89
BS	11.61	67.52	23.1	21.13	20.46
Burdock	10.82	38.71	21.69	28.17	8.02
Circe	32.87	29.61	77.32	44.04	22.55
copia	3.13	34.19	17.55	43.16	3.49
diver	10	56.14	**19.4**	18.52	4.66
Dm88	4.19	23.8	3.4	1.19	2.8
Doc	30.18	63.24	35.13	83.16	21.52
Doc2-element	3.04	25.31	8.65	4.36	4.5
F-element	27.08	41.36	91.09	11.75	25.28
FB	-0.54	-0.32	-0.76	2.68	-0.53
flea	9.61	18.56	**42.64**	14.47	10.25
G2	7.82	25.98	40.8	23.39	11.71
G6	2.21	29.6	20.31	13.8	10.83
gypsy6	9.22	8.81	4.27	5.12	10.25
HMS-Beagle	13.18	18.04	27.08	23.14	12.35
HMS-Beagle2	3.81	2.57	0.89	15.04	0.78
hobo	1.8	27.38	0.48	0.73	1.01
hopper	2.5	96.92	8.33	11.55	0.53
I-element	1.46	23.39	8.66	1.48	5.7
Idefix	7.32	19.15	5.1	4.68	2.25
invader3	16.23	55.52	21.18	53.34	9
invader4	2.71	4.67	2.16	15.4	1.05
invader6	25.47	26.3	18.35	2.76	5.11
Ivk	42.31	12.66	94.54	4.26	17.97
jockey	41.75	68.53	**24.81**	15.79	16.92
Juan	5.97	16.48	4.9	17.59	3.93
Max-element	14.52	22.52	50.95	8.78	23.89
McClintock	6.04	11.94	8.72	3.04	2.42
mdg1	1.63	3	10.34	8.09	0.89
mdg3	5.84	11.03	13.58	18.22	7.17
micropia	13.43	24.48	22.81	5.11	19.15
NOF	0.93	-0.49	-0.26	-0.13	3.15
opus	39.48	99.41	59.12	115.53	31.04
P-element	NA	40.73	NA	NA	15.24
pogo	3.16	7.56	3.84	2.23	1.13
Quasimodo	1.33	7.49	24.41	32.94	20.02
R1A1-element	39.52	39.46	93.09	75.23	63.84
R2-element	21.58	**-0.53**	**3.07**	**0.35**	**1.71**
roo	13.43	30.6	9.04	19.81	11.56
rover	1.83	21.69	18.44	10.28	12.72
Rt1a	28.28	34.31	36.74	33.95	31.76
Rt1b	24.93	9.69	28.68	58.05	17.79
S-element	-0.06	21.17	10.75	14.8	-0.66
springer	11.84	2.71	2.01	-0.07	37.91
Stalker	11.47	9.2	14.76	2.03	11.8
Stalker2	0.44	5.94	2.98	0.52	0.04
Stalker4	8.17	11.48	9.93	16.86	5.78
Tirant	NA	11.73	**10.14**	**0.86**	3.36
transib2	4.4	39.57	4.94	5.71	3.19
Transpac	5.05	36.17	10.59	7.56	4.61
X-element	21.93	65.76	15.82	45.37	36.25

Table S5: TE families considered for analyses in this work. TE families are sorted by their average population frequency based on previous estimates.

TE family	seqID	order	population frequency (%)
P-element	PPI251	TIR	2.9
Tirant	TIRANT	LTR	3.2
R2-element	DMRER2DM	non-LTR	3.4
Stalker	STALKER	LTR	3.5
blood	BLOOD	LTR	3.9
copia	DMCOPIA	LTR	3.9
mdg1	DMRTMGD1	LTR	3.9
412	412	LTR	4
rover	ROVER	LTR	4
springer	SPRINGER	LTR	4.2
GATE	DME010298	LTR	4.3
Stalker2	STALKER2	LTR	4.3
Transpac	AF222049	LTR	4.4
mdg3	DMMDG3	LTR	4.4
jockey	DMLINEJA	non-LTR	4.6
opus	OPUS	LTR	4.6
Circe	CIRC	LTR	4.7
Doc	DMW1DOC	non-LTR	4.8
Burdock	DMU89994	LTR	5
Max-element	DME487856	LTR	5
R1A1-element	DMRER1DM	non-LTR	5
diver	Tinker	LTR	5
gypsy6	GYPSY6	LTR	5
Juan	JUAN	non-LTR	5.1
invader6	INVADER6	LTR	5.3
F-element	F	non-LTR	5.5
pogo	DMPOGOR11	TIR	5.6
HMS-Beagle	Beagle	LTR	6
Dm88	DM88	LTR	6.5
G2	G2	non-LTR	6.5
McClintock	McCLINTOCK	LTR	6.5
hopper	DMTRDNA	TIR	6.6
roo	DM_ROO	LTR	6.6
NOF	FB	TIR	6.8
hobo	DMHFL1	TIR	6.9
3S18	DM23420	LTR	7
flea	DMBLPP	LTR	7.1
HMS-Beagle2	Beagle2	LTR	7.2
I-element	DMIFACA	non-LTR	7.8
17.6	DMIS176	LTR	7.9
accord	ACCORD	LTR	8
297	DMIS297	LTR	8.1
Quasimodo	QUASIMODO	LTR	8.1
Bari1	DMBARI1	TIR	8.9
Ivk	IVK	non-LTR	8.9
accord2	QBERT	LTR	9.2
Rt1a	DME278684	non-LTR	9.7
Idefix	DME9736	LTR	9.8
Stalker4	STALKER4	LTR	10
FB	DMTNFB	Foldback	10.2
Rt1b	RT1B	non-LTR	10.3
BS	BS	non-LTR	11.6
invader4	INVADER4	LTR	13.3
G6	G6_DM	non-LTR	13.4
invader3	INVADER3	LTR	16.4
X-element	ROXELEMENT	non-LTR	17
micropia	DMDM11	LTR	18.1
Doc2-element	DOC2	non-LTR	20.4
transib2	TRANSIB2	TIR	21.1
1731	DMTN1731	LTR	23
S-element	DM33463	TIR	23.2
